# The TENT5A-ATXN2 axis modulates germline and somatic cell survival during heat stress

**DOI:** 10.1038/s41419-026-09086-1

**Published:** 2026-07-14

**Authors:** Ru-pin Alicia Chi, Ankit Gupta, Yin Li, Marine Baptissart, Dongwon Lee, Brian N. Papas, Marcos Morgan

**Affiliations:** 1 Reproductive and Developmental Biology Laboratory, National Institute of Environmental Health Sciences, National Institutes of Health, Durham, NC USA; 2 Integrative Bioinformatics, Biostatistics and Computational Biology Branch, National Institute of Environmental Health Sciences, National Institutes of Health, Durham, NC USA

**Keywords:** Spermatogenesis, Male factor infertility, RNA modification, Apoptosis

## Abstract

Stress conditions elicit the formation of different kinds of stress granules (SGs). Although male germ cells are particularly sensitive to heat stress, the composition of heat-induced SGs in the testis remains poorly characterized. Here, we show that ATXN2 is a main component of heat-induced SGs in male germ cells and identify a unique population of ATXN2-positive SGs in leptotene and zygotene spermatocytes lacking classical SG markers. The terminal nucleotidyltransferase 5A (TENT5A) is a cytoplasmic poly(A) polymerase that extends the poly(A) tails of mRNAs. *Tent5a* mRNA expression increases when somatic and germ cells are exposed to 42 °C, but not upon exposure to other stressors. In vitro, TENT5A extends the poly(A) tail of *Atxn2* transcripts, stabilizing them while repressing their translation. In heat-stressed testes, TENT5A depletion increases ATXN2-positive granules and reduces apoptosis in late pachytene spermatocytes. Thus, enhanced stress resilience in *Tent5a*-depleted male germ cells preserves spermatogenesis and rescues fertility following heat exposure.

## Introduction

When exposed to environmental stressors, cells must mount rapid and tailored responses to ensure adaptation, survival and continued function. One common response is the formation of stress granules (SGs), which modulates mRNA translation [1]. SGs store mRNAs in a translationally repressed state and unlike P-bodies, lack decapping enzymes that trigger transcript decay [2]. Consequently, SG-associated mRNA can be rapidly re-engaged for translation once stress subsides [3, 4]. G3BP stress granule assembly factor 1 (G3BP1), a core SG component, coordinates multivalent protein-RNA interactions that drive SG assembly and sequester transcripts from the cytoplasm [5]. However, SG composition and transcript exchange are stimulus-dependent, and certain factors can compensate for G3BP1 loss – for example, under osmotic or heat stress [6].

Ataxin 2 (ATXN2) is an RNA-binding protein commonly found in SGs [7]. Structurally, it contains Lsm and Lsm-associated domains that mediate RNA binding, as well as a PAM2 domain that enables interaction with poly(A)-binding proteins. Most of ATXN2, however, consists of intrinsically disordered regions [8], which facilitate multivalent interactions that drive granule assembly. In yeast, the ATXN2 orthologue promotes mRNA poly(A) tail extension, a feature linked to transcript stabilization [9, 10]. Members of the terminal nucleotidyltransferase 5 family (TENT5A, TENT5B, TENT5C and TENT5D) similarly elongate the poly(A) tail of select mRNAs essential for bone formation and fertility [11–14]. In particular, TENT5A, TENT5C and TENT5D have been associated with male infertility [11, 12, 14]. Poly(A) tail length extension generally enhances transcript stability and, depending on the context, can modulate translational activity [15, 16]. Because non-templated poly(A) modification can rapidly alter protein output without requiring de novo transcription, it represents an ideal mechanism for stress adaptation. Given the high sensitivity of male germ cells to heat stress, TENT5s may contribute to their heat stress response through regulation of SGs. However, the role of TENT5 proteins in SG function, particularly in the context of heat stress, remains poorly understood.

The testis is the most heat-sensitive organ in the body, and hence in mammals, it is located outside the abdominal cavity to maintain a temperature 2–7 **°**C below core body temperature [17]. Sustained exposure of the testis to 37 **°**C is incompatible with fertility, as seen in individuals with undescended testes [18]. Even transient temperature elevations from occupational or recreational activities can impair sperm production [19–22]. In response to heat, G3BP1-positive granules form in pachytene spermatocytes (SPCs), while deleted in azoospermia like (DAZL)-positive granules assemble in differentiated spermatogonia (SPG), preleptotene and pachytene SPCs [23]. Despite these observations, the mechanisms regulating the heat stress response in the mammalian testis remain poorly characterized.

Here, we show that TENT5A mRNA is upregulated specifically upon hyperthermia across multiple cell lines, including HEK293, HeLa, 17Cl1 and mouse embryonic fibroblast (MEF), as well as in the testis. Through poly(A) elongation, TENT5A stabilizes ATXN2 mRNA while reducing its translation, and via complex formation with ATXN2 protein, further reduces ATXN2 protein abundance. In the testis, ATXN2-positive granules are more prevalent than SGs containing G3BP1, poly(A) binding protein, cytoplasmic 1 (PABPC1) or cytotoxic granule-associated RNA binding protein 1 (TIA1), as well as the germline-specific DAZL granules. TENT5Adepletion increases ATXN2-positive SG formation after scrotal heat exposure, reducing cell type-specific apoptosis. In summary, TENT5A is a heat-specific responder that downregulates ATXN2 protein and suppresses SG formation, thereby modulating male germ cell fate under heat stress.

## Results

### *TENT5A* expression increases specifically in response to heat stress in a dose-dependent manner

**Fig. 1:**
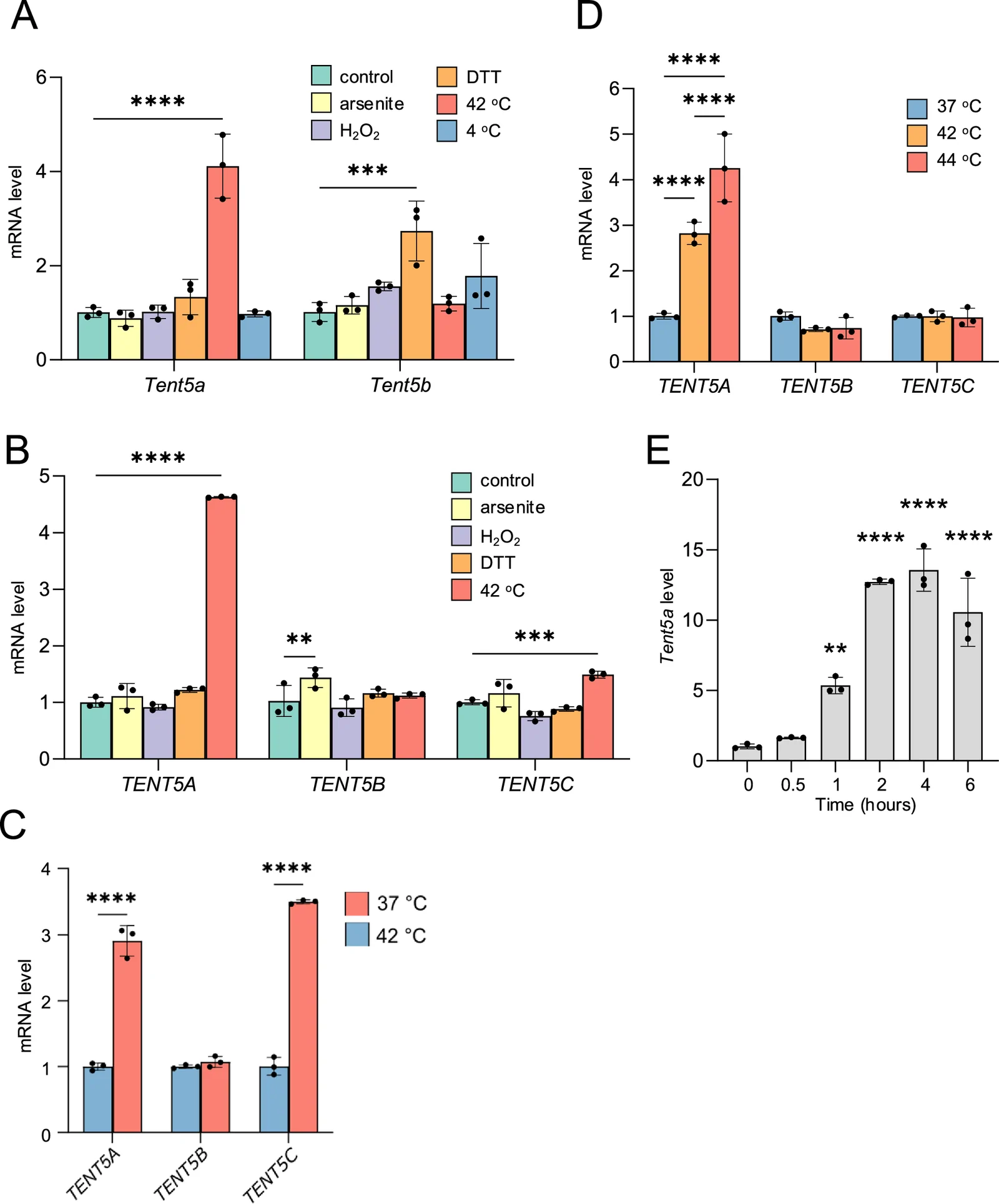
*TENT5A* mRNA is robustly upregulated by heat stress in multiple cell lines. **A** Bar plot of *Tent5a* and *Tent5b* mRNA levels in 17Cl1 cells upon exposure to sodium arsenite, H_2_O_2_, DTT, 42 °C, and 4 °C, as determined by qPCR. Control cells were untreated and cultured at 37 °C. **B** Bar plot of *TENT5A*, *TENT5B* and *TENT5C* mRNA levels in HEK293 cells upon exposure to sodium arsenite, H_2_O_2_, DTT, and 42 °C, as determined by qPCR. Control cells were untreated and cultured at 37 °C. **C** Bar plot of *TENT5A*, *TENT5B*, and *TENT5C* mRNA levels in HeLa cells upon incubation at 37 °C or 42 °C, as determined by qPCR. **D** Bar plot of *TENT5A*, *TENT5B*, and *TENT5C* mRNA levels in HEK293 cells incubated at 37 °C, 42 °C and 44 °C, as determined by qPCR. **E** Bar plot of *Tent5a* mRNA levels in 17Cl1 cells incubated at 42 °C for 0, 0.5, 1, 2, 4, and 6 h, as determined by qPCR. Results are presented as mean ± SD from three biological replicates. Statistical significance was determined by two-tailed Student’s *t*-test: **p < 0.01; ***p < 0.001; ****p < 0.0001.

To understand whether cytoplasmic polyadenylation plays a role in the cellular stress response, we examined the mRNA expression of the non-canonical cytoplasmic poly(A) polymerases encoded by the *TENT5* genes (*TENT5A*, *TENT5B*, *TENT5C*, and *TENT5D*) upon exposure to different environmental insults. We first subjected 17Cl1, a mouse fibroblast cell line that expresses only *Tent5a* and *Tent5b* (Table S1), to oxidative stress (sodium arsenite and H₂O₂), endoplasmic reticulum (ER) stress (DTT), and temperature stress (heat and cold shock at 42 °C and 4 °C, respectively). *Tent5a* transcripts were upregulated by 4-fold specifically in response to high temperature (Fig. 1A). Additionally, *Tent5b* showed a ~ 3-fold increase in response to DTT (Fig. 1A). To confirm that the cells were indeed stressed under the applied conditions, we examined SG formation using the SG marker, G3BP1 (Fig. S1A). Oxidative, endoplasmic reticulum, and heat stress all induced SG formation, whereas cold shock did not (Fig. S1A). Since stress responses can vary across cell types and conditions, we further examined *Xbp1* mRNA splicing as a marker of the unfolded protein response (UPR), which is linked to cellular stress [24]. As expected, DTT treatment caused a marked increase in the ratio of spliced *Xbp1* (*Xbp1-s*) to unspliced *Xbp1* (*Xbp1-u*). Both heat and cold shock also increased *Xbp1* splicing, indicating that both stressors activated the UPR (Fig. S1B). The lack of correlation between SG formation and *Xbp1* splicing confirms that cells respond uniquely to different stressors. In this context, *Tent5a*’s induction is heat-specific.

Next, we examined whether *TENT5A* responds to heat stress in different cell lines. HEK293 cells, which do not express *TENT5D*, also showed increased *TENT5A* expression specifically in response to heat stress (Fig. 1B, Table S1). Heat also induced *TENT5C* expression, although the magnitude of change was smaller (Fig. 1B). Additionally, a small but significant upregulation of *TENT5B* was also observed for sodium arsenite-treated HEK293 cells (Fig. 1B). However, the lack of consistency in DTT-induced and sodium arsenite-induced *Tent5b* response between cell lines indicated that this is likely cell line-specific. We further examined whether *Tent5* members respond to heat stress in HeLa cells, which do not express *TENT5D* (Table S1). In HeLa cells, heat exposure induced *TENT5A* expression, while *TENT5B* showed no response (Fig. 1C). Interestingly, *TENT5C* showed a similar level of induction as *TENT5A* (Fig. 1C). The consistent increase in *TENT5A* mRNA levels across human and mouse cell lines suggests that this is a general heat-response mechanism. Furthermore, *TENT5A’s* response was dose-dependent, as greater induction was observed at 44 °C compared to 42 °C in HEK293 cells (Fig. 1D). Notably, *TENT5C* did not show a pronounced increase under the same conditions (Fig. 1B, D), indicating that *TENT5C* is not consistently responsive to heat. Finally, we examined changes in *Tent5a* expression over six hours of heat exposure. In 17Cl1 cells, *Tent5a* expression peaked at 2–4 h and began to decline by 6 h, although levels remained higher than baseline (Fig. 1E). In sum, the expression of *TENT5A* mRNA is robustly and specifically increased upon heat exposure across different cell types.

### TENT5A suppresses the heat-induced stress response and promotes cell death

**Fig. 2:**
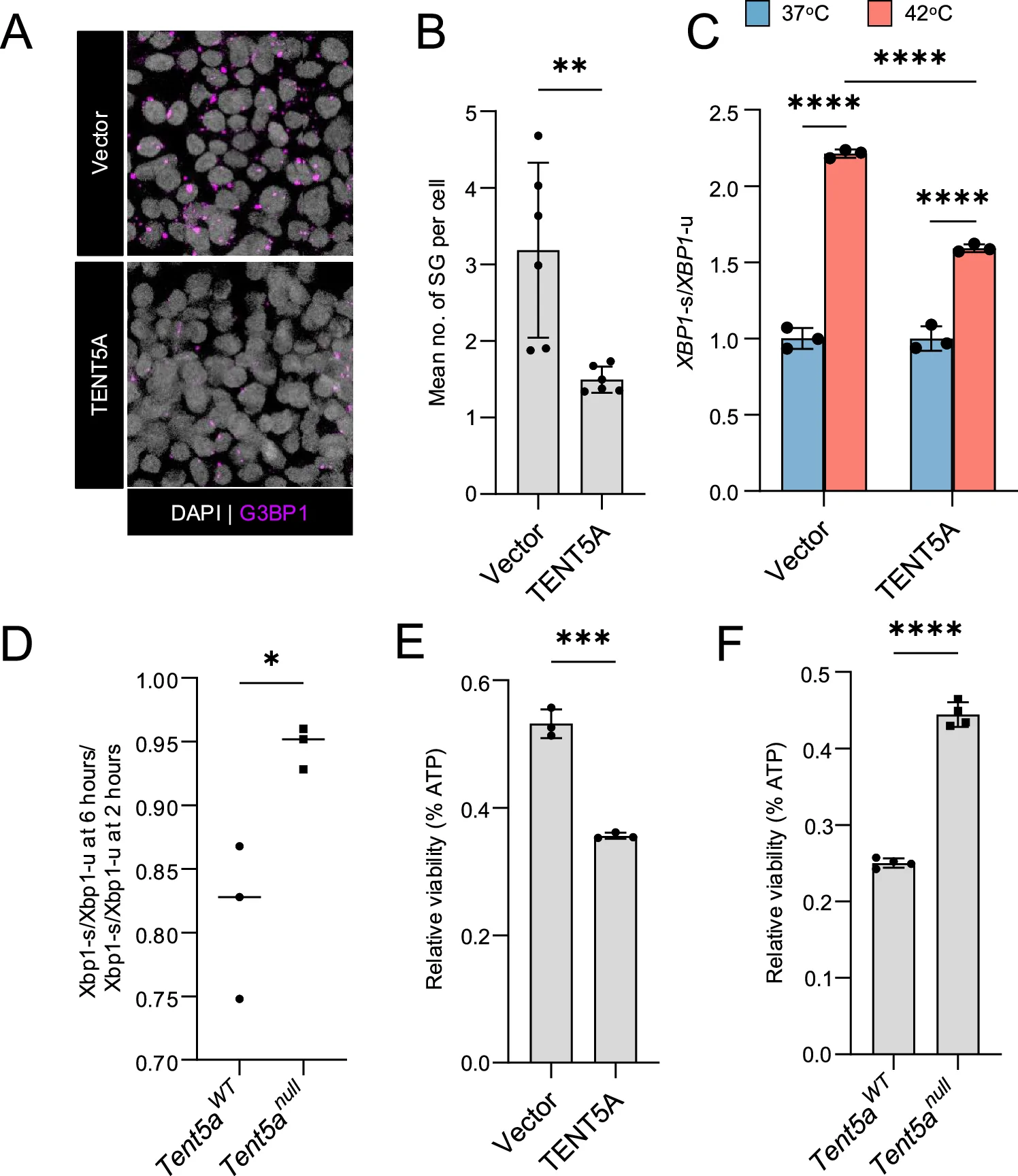
TENT5A suppresses the heat-induced stress response and promotes cell death. **A** Confocal images of HEK293 cells transfected with FHpHG-*Tent5a* (TENT5A) or FHpHG (Vector), incubated at 42 °C and stained with DAPI (white) and anti-G3BP1 (purple). Scale bar = 20 µm. The experiment was repeated three times. **B** Bar plot showing the mean number of SGs per cell in TENT5A-overexpressing and control HEK293 cells after heat exposure. **C** Bar plot showing the ratio of spliced *XBP1*-s to *XBP1*-u in control and TENT5A-overexpressing HEK293 cells incubated at 37 °C or 42 °C. **D** Dot plot showing the ratio of *Xbp1*-s to *Xbp1*-u at 6 h relative to 2 h of 42 °C incubation in *Tent5a*^*WT*^ and *Tent5a*^*null*^ MEF cells. The center value indicates the median ratio. **E** Bar plot showing ATP levels as percentage in HEK293 cells transfected with FHpHG-*Tent5a* (TENT5A) or FHpHG (Vector) after 16 h of incubation at 42 °C relative to 37 °C. **F** Bar plot showing ATP levels as percentage in *Tent5a*^*WT*^ and *Tent5a*^*null*^ MEF cells after 6 h of incubation at 42 °C relative to 37 °C. (*n* = 4, biological replicates). Results are presented as mean ± SD from three biological replicates, unless otherwise indicated. Statistical significance was determined by Student’s *t* test (**B**, **D**, **E**, **F**) and two-way ANOVA (**C**): *p < 0.05; **p < 0.01; ***p < 0.001; ****p < 0.0001.

Given the specific regulation of TENT5A during heat exposure, we speculated that TENT5A is involved in the heat-induced stress response. To test this hypothesis, we examined how altered TENT5A levels affect various cellular responses to heat stress. First, we generated a construct that constitutively expresses TENT5A under the control of the CMV promoter. TENT5A was tagged at its N-terminus with a FLAG tag, two HA tags, a human rhinovirus cleavage site, a six-histidine affinity tag, and an enhanced GFP gene (*FHpHG-Tent5a*). HEK293 cells were transfected with either the FHpHG tag alone (vector), or FHpHG-*Tent5a* (TENT5A). Overexpression was confirmed by detection of the HA tag (Fig. S2A). We first compared SG formation in control and TENT5A-overexpressing HEK293 cells. Transfected cells were incubated at 42 °C for 1 h, and SGs were visualized using the SG marker G3BP1. Heat-exposed TENT5A-overexpressing cells showed fewer SGs compared to heat-exposed control cells (Fig. 2A, B), suggesting that TENT5A suppresses SG formation. We next assessed *Xbp1* splicing after heat exposure in control and *Tent5a*-overexpressing cells. *Tent5a* overexpression resulted in reduced *Xbp1* splicing compared to control cells (1.6-fold vs. 2.2-fold increase, respectively; Fig. 2C). To further examine stress response dynamics, we quantified *Xbp1* splicing in three *Tent5a*^*WT*^ and three *Tent5a*^*null*^ MEF cell lines at 2 and 6 hours post-heat exposure (Fig. S2B, C). While *Xbp1* splicing declined from 2 to 6 h in both WT and TENT5A-depleted MEF cells, the decline was significantly less pronounced in *Tent5a*^*null*^ MEF cells (Fig. 2D). These findings suggest that TENT5A represses the cellular response to heat stress, as increased levels of TENT5A led to reduced SG formation and *Xbp1* splicing.

Finally, because SGs have been reported to either promote or repress apoptosis under different stress conditions [25–27], we assessed post-heat cell survival in TENT5A-overexpressing and TENT5A-depleted cells. We used the CellTiter-Glo Luminescence Assay Kit, which measures metabolic state via ATP levels, a proxy for cell viability. TENT5A expression inversely correlated with cell survival after heat exposure: TENT5A overexpression reduced viability, whereas TENT5A depletion improved survival (Fig. 2E, F). Together, these findings indicate that under the heat stress conditions studied, TENT5A functions to repress the stress response and promote cell death.

### TENT5A polyadenylates ATXN2 mRNA and interacts with ATXN2 protein to downregulate its expression

**Fig. 3:**
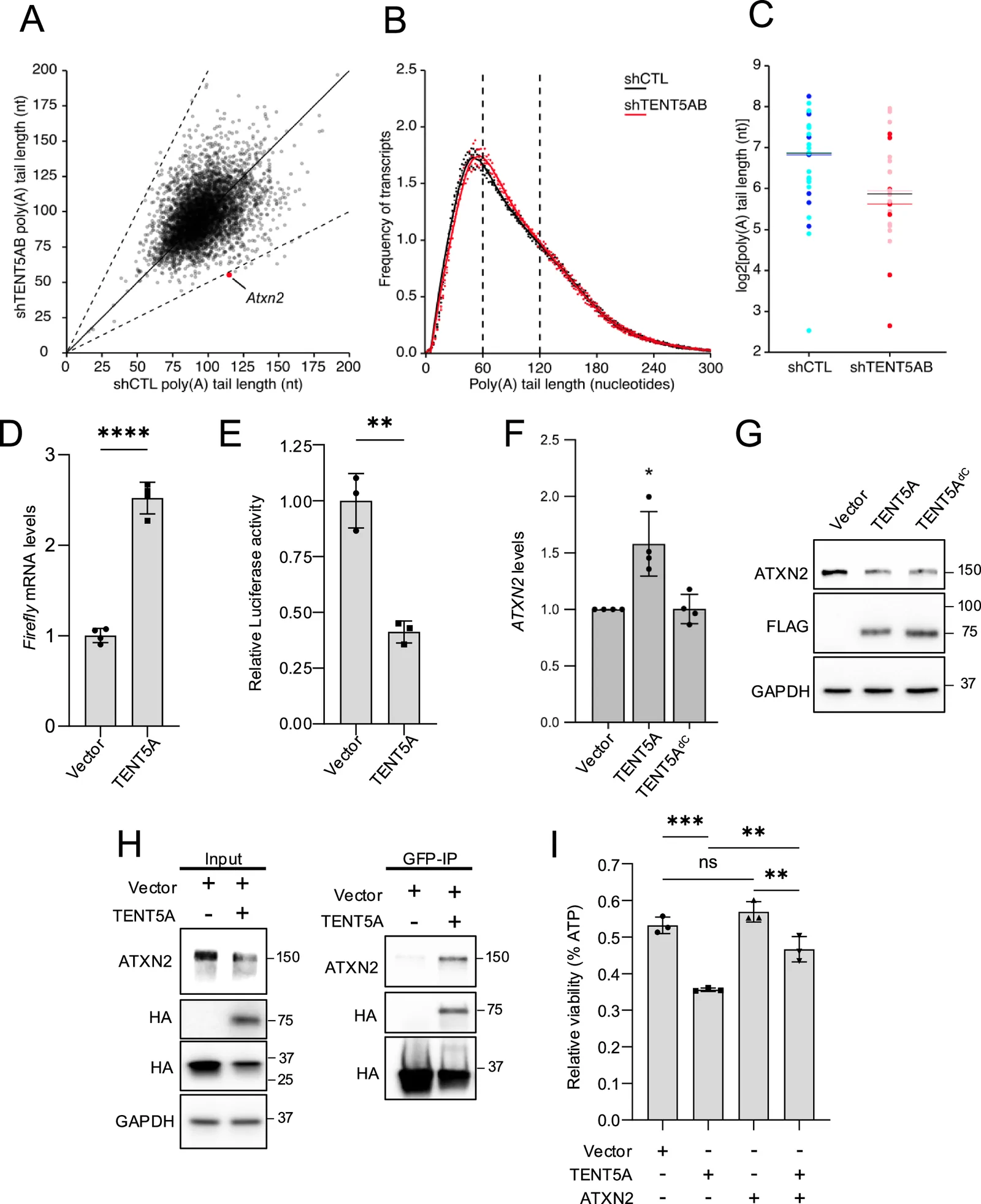
TENT5A downregulates ATXN2 expression levels. **A** Scatter plot showing the median poly(A) tail length (nt) of transcripts from stable 17Cl1 cells transfected with shRNAs targeting *Tent5a* and *Tent5b* (sh*Tent5a*/*b*, *n* = 2 independent clones) or a control shRNA (shCTL, *n* = 3 independent clones). The red dot indicates the median poly(A) tail length of the *Atxn2* transcripts in sh*Tent5a*/*b* and shCTL cells. **B** Density plot of poly(A) tail lengths of transcripts from 17Cl1 cells transfected with sh*Tent5a*/*b* (shTENT5A/B, red) or control shRNA (shCTL, black). **C** Dot plot showing the median poly(A) tail lengths of *Atxn2* transcripts in sh*Tent5a*/b-treated and shCTL-treated 17Cl1 cells. The median poly(A) tail for each replicate is indicated in different shades of blue and red for the shCTL and shTENT5A/B conditions, respectively. **D** Bar plot showing luciferase mRNA expression in HEK293 cells transfected with the luciferase reporter containing the *Atxn2* 3’UTR, together with either the vector or the TENT5A-overexpression construct. The Firefly mRNA levels were normalized to the respective Renilla mRNA levels. (*n* = 4, biological replicates). **E** Bar plot showing luciferase activity in HEK293 cells transfected with a luciferase reporter containing the *Atxn2* 3’UTR, together with either the vector or the TENT5A-overexpression construct. The Firefly luminescence signals were normalized to the respective Renilla luminescence signals to account for transfection efficiency, then normalized again to the control 3’ UTR. **F** Bar plot showing *ATXN2* mRNA levels in HEK293 cells transfected with FHpHG-Tent5a (TENT5A), FHpHG-Tent5a^dC^ (TENT5A^dC^), or FHpHG (vector), as determined by qPCR. (*n* = 4, biological replicates). **G** Western blot of extracts from HEK293 cells overexpressing TENT5A, TENT5A^dC^, or vector control. Blots were probed with anti-ATXN2, anti-FLAG, and anti-GAPDH antibodies, with the latter being the loading control. Molecular weight markers in kDa are indicated on the right. **H** Immunoprecipitation of extracts from HEK293 cells overexpressing TENT5A or vector control using GFP nanobody. Western blots of input samples (left) and immunoprecipitates (right) were probed with anti-ATXN2 and anti-HA tag at 75 and 37 kDa for detection of FHpHG-Tent5a and FHpHG, respectively. GAPDH was probed to confirm equal loading in each lane, and the molecular weight markers in kDa are indicated on the right. **I** Bar plot showing relative ATP levels as a proxy for cell viability in control, TENT5A-, ATXN2-, or TENT5A and ATXN2-overexpressing cells following 16 hours incubation at 42 °C, normalized to 37 °C conditions. Quantitative results are presented as mean ± SD of three replicate values, unless otherwise indicated. Student’s *t* test was used to compare each condition to the control condition, **p < 0.01; ***p < 0.001; ****p < 0.0001.

To understand the mechanisms by which TENT5A regulates the cellular response to heat stress, we next sought to identify its targets. As the TENT5 family members exhibit functional redundancy to some degree [11, 12], we established stable *Tent5a/Tent5b* double knockdown 17Cl1 cells (Fig. S3A, Table S1). We then profiled the global transcript poly(A) tail lengths using Nanopore-based direct RNA sequencing. No global shortening of poly(A) tails was observed in the *Tent5a/b* knockdown cells (Fig. 3A, B), suggesting that the TENT5 proteins target specific transcripts, which is consistent with observations in other systems [11, 12, 16, 28]. Among transcripts with shorter poly(A) tails in the absence of TENT5A/B, we focused on *Atxn2* because it showed ~50% reduction in tail length (Fig. 3C) and is a known regulator and component of SGs [7, 29]. We first examined whether SGs formed in our heat-shocked cell lines contained ATXN2. Co-labeling of G3BP1 and ATXN2 in heat-exposed 17Cl1 and other cells showed complete colocalization, confirming that these SGs are ATXN2-positive (Fig. S3B).

The 3’ untranslated region (3’UTR) of a transcript is known to regulate poly(A) tail length by interacting with RNA-binding proteins which then recruit poly(A)-modifying enzymes, such as the TENT proteins [30–33]. Hence, to investigate whether TENT5A regulates *ATXN2* expression through its 3’UTR, we cloned *ATXN2* 3’UTR downstream of a luciferase reporter gene. We observed that while the *Firefly* luciferase mRNA level increased, the luciferase activity –a readout of luciferase protein expression– was reduced in *TENT5A*-overexpressing cells compared to control cells (Fig. 3D, E). These findings support that TENT5A regulates ATXN2 at least partially through its transcript’s 3’UTR.

To further evaluate whether TENT5A represses ATXN2 via poly(A) tail regulation, we generated a catalytically inactive TENT5A mutant (FHpHG-*Tent5a*^*DeltaCat*^, hereafter referred to as *Tent5a*^*dC*^) by substituting aspartates at positions 144 and 146 with alanines, abolishing its poly(A) polymerase activity [11]. Overexpression of TENT5A, but not TENT5A^dC^, led to an increase in *ATXN2* transcript levels, indicating that mRNA stabilization requires TENT5A’s catalytic activity (Fig. 3F). However, ATXN2 protein levels were similarly reduced upon overexpression of both TENT5A and TENT5A^dC^ (Fig. 3G), suggesting additional regulatory mechanisms. A literature search revealed that both TENT5A and ATXN2 directly interact with ATXN1 [34], raising the possibility that TENT5A regulates ATXN2 via protein complex formation. To test this, we transfected HEK293 cells with a FHpHG-TENT5A-expressing construct and performed GFP immunoprecipitation. TENT5A pulled down endogenous ATXN2 (Fig. 3H), indicating that both proteins can be present in the same protein complex.

Finally, we examined the functional consequences of ATXN2 on cell viability under heat stress. While ATXN2 overexpression alone had little impact on survival following heat shock (Fig. 3I), co-overexpression of TENT5A and ATXN2 partially rescued the heat-induced cell death observed in TENT5A-overexpressing cells (Fig. 3I). Together, these findings support an antagonistic relationship between ATXN2 and TENT5A in modulating cell fate under hyperthermia.

### ATXN2-positive heat-induced SGs are present in diverse male germ cell types

**Fig. 4:**
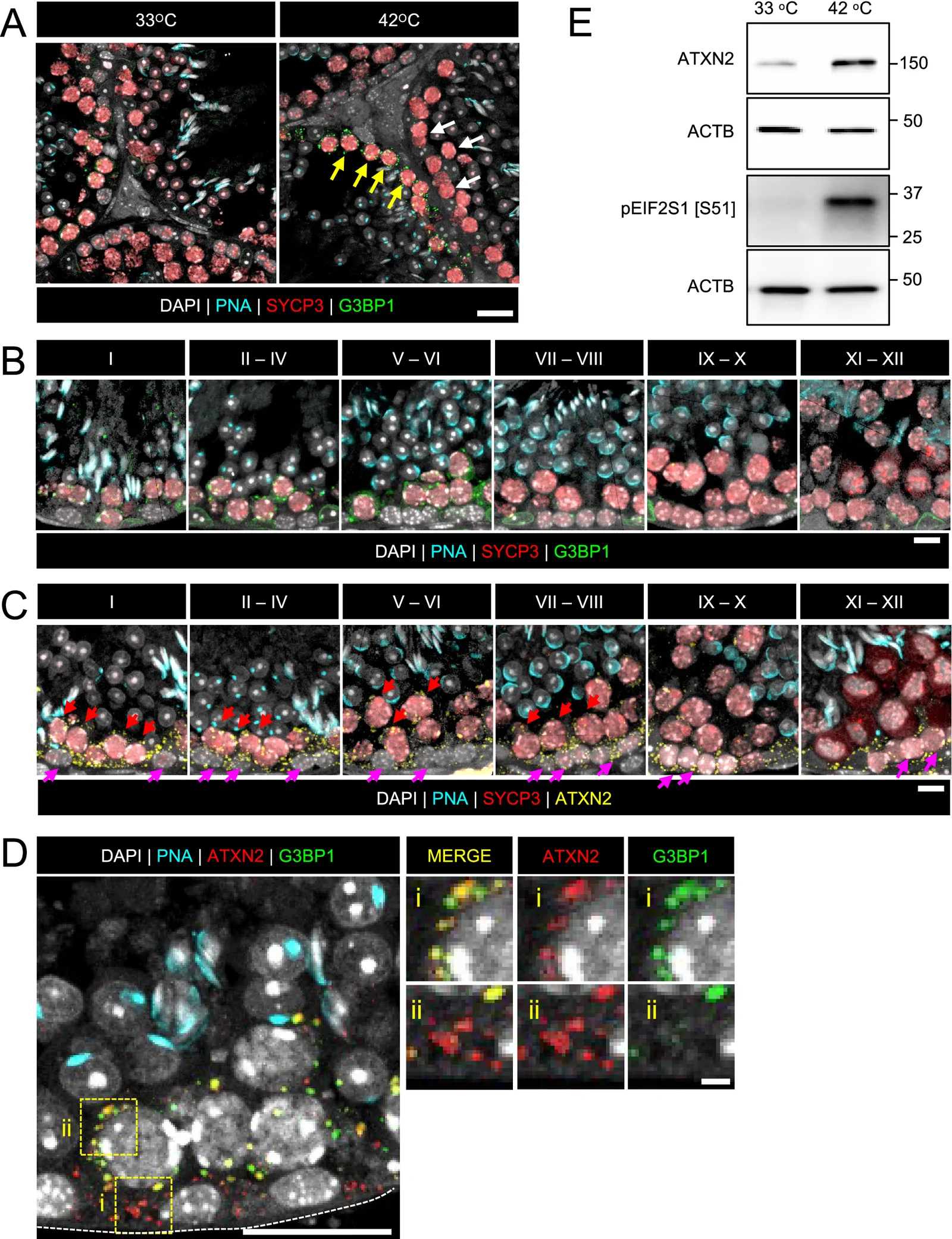
ATXN2 forms heat-induced stress granules in spermatogonia and spermatocytes. **A** Confocal images of testicular cross-sections from WT mice following scrotal exposure to 33 °C or 42 °C. Sections were immunostained with anti-G3BP1 antibody (green) and anti-SYCP3 antibody (red). Nuclei were counterstained with DAPI (white), and acrosomes were stained with PNA (cyan). The yellow and white arrows indicate SPCs with or without SGs after heat exposure, respectively. Scale bar = 20 µm. **B**, **C** Confocal images of testicular cross-sections from WT mice after 20 min of scrotal exposure to 42 °C. Acrosomes and SPCs were labeled with PNA (cyan) and SYCP3 (red), respectively. G3BP1 (green, **B**) and ATXN2 (yellow, **C**) are shown in separate panels. Representative seminiferous tubule stages I, II–IV, V–VI, VII–VIII, IX–X, and XI–XII are displayed. Purple arrows indicate intermediate SPGs, B SPGs, and pre-pachytene SPCs; red arrows indicate pachytene SPCs. Scale bar = 10 µm. **D** Confocal images of a testicular cross-section from WT mice following 20 min of scrotal exposure to 42 °C. ATXN2 (red) and G3BP1 (green) were detected by immunofluorescence. Acrosomes and nuclei were stained with PNA (cyan) and DAPI (white), respectively. Enlarged regions (i) and (ii) are shown on the right. Scale bars: 20 µm (left), 2 µm (right). Confocal experiments (**A**–**D**) were repeated with different mice three times. **E** Western blots of testicular extracts from WT mice exposed to scrotal temperatures of 33 °C or 42 °C. Blots were probed with anti-ATXN2 and anti-pEIF2S1 antibodies. ACTB was probed to verify protein loading. Molecular weight markers (kDa) are indicated on the right.

The male germline is highly thermosensitive, yet how it manages and responds to heat stress remains incompletely understood. To investigate whether TENT5A and ATXN2 are involved in this process, we first characterized the germline response to heat stress using WT mice. Localized scrotal heating was applied by submerging the lower body of the animals in a water bath maintained at 33 °C (control) or 42 °C (heat-exposed). Post-exposure, SG formation was assessed by G3BP1 immunostaining in testicular sections. SGs were predominantly detected in SYCP3-positive pachytene SPCs, although not all SPCs formed granules (Fig. 4A, yellow vs. white arrows).

To define the cell types that form heat-induced SGs, we examined G3BP1 expression in staged seminiferous tubules using SYCP3 and peanut agglutinin (PNA) staining. Numerous SGs were observed in stage I–VI pachytene SPCs, with few in stages VII–VIII, and none in later-stage SPCs or spermatids (Fig. 4B). We next assessed whether ATXN2 is a component of the germline SGs. Unlike G3BP1-positive SGs, ATXN2-positive SGs were found in intermediate and B SPG, preleptotene to zygotene SPC as well as pachytene SPCs from stages I to VII/VIII (Fig. 4C, purple and red arrows, respectively). Co-labeling of ATXN2 and G3BP1 revealed that the pachytene SPC granules exhibited either partial or complete colocalization of the two proteins (Fig. 4D, insets i), while in the B SPG, SGs contained predominantly or exclusively ATXN2 (Fig. 4D, insets ii). These observations suggest that heat-induced germline SGs are more complex and dynamic than in somatic cells (Fig. S3B and Fig. 4D).

Prompted by the presence of ATXN2-positive, G3BP1-negative SGs in the differentiating SPG and preleptotene, leptotene, and zygotene SPCs, we examined other known germline SG markers in these cells [23, 35]. Similar to G3BP1, PABPC1 and TIA1 were restricted to pachytene SPC SGs and absent from all other populations (Fig. S4A and S4B). On the other hand, DAZL-containing SGs were present in the intermediate and B SPG; preleptotene SPCs; and stage I–VII/VIII pachytene SPCs as previously observed (Fig. S4C)[23]. None of these SG components were detected in the post-meiotic spermatids (Figs. 4A–D and S4A–C). Together, these findings show that heat-induced SGs form in germ cells spanning SPG to mid-pachytene SPCs, and among all markers examined, ATXN2 spanned the widest range of germ cell types and was exclusively present in leptotene and zygotene SPCs. A summary of SG marker expression across cell types is shown in Fig. S4D. Given its prominence, we examined whether testicular ATXN2 protein levels are heat-responsive. Heat exposure induced EIF2S1 phosphorylation and elevated ATXN2 expression in testicular lysates (Fig. 4E). These data collectively demonstrate that ATXN2 is a widespread component of heat-induced SGs in the male germline.

### *Tent5a* is expressed in the testis and modulates the heat stress response

**Fig. 5:**
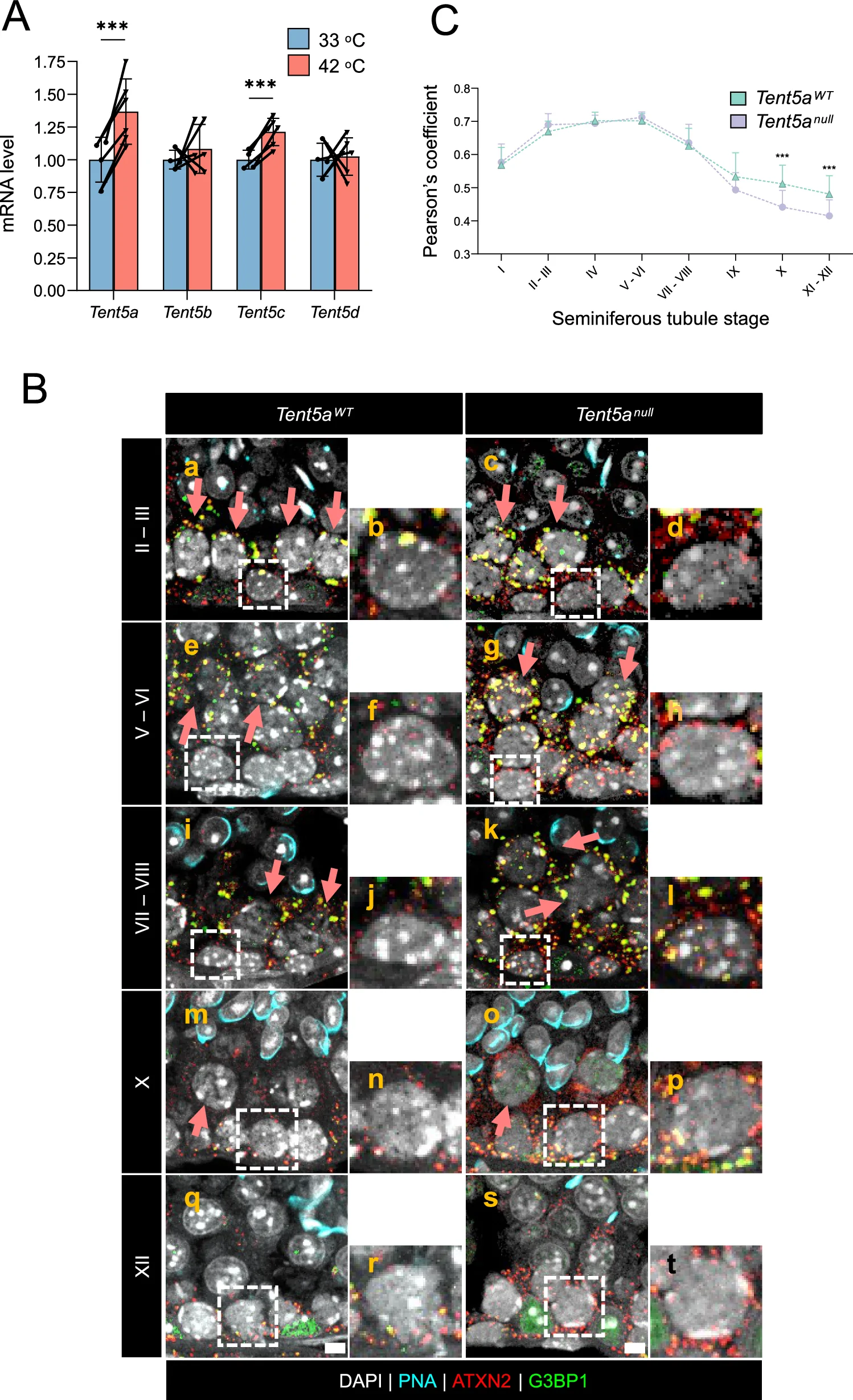
TENT5A-deficient animals exposed to scrotal heat show increased ATXN2-positive granules. **A** Bar plots showing *Tent5a*, *Tent5b*, *Tent5c*, and *Tent5d* mRNA levels in whole testis extracts from WT mice exposed to scrotal temperatures of 33 °C (blue) or 42 °C (red), as determined by qPCR. **B** Confocal images of testicular cross-sections from stages II–III, V–VI, VII–VIII, IX–X, and XII in *Tent5a*^*WT*^ and *Tent5a*^*null*^ mice. Insets highlight intermediate SPG (b, d), B SPG (f, h), preleptotene SPC (j, l), leptotene SPC (n, p), and zygotene SPC (r, t). Pink arrows indicate pachytene SPCs in the main images. Testes were collected within 30 min after 20 min of scrotal exposure to 42 °C. Sections were immunostained with anti-ATXN2 (red), anti-G3BP1 (green); acrosomes were stained by PNA (cyan), and nuclei were counterstained with DAPI (white). Scale bars, 5 µm (main images) and 2 µm (insets). The experiment was repeated with different control and experimental mice pairs twice. **C** Line graph showing Pearson correlation coefficients indicating ATXN2-G3BP1 colocalization across seminiferous tubule stages in *Tent5a*^*WT*^ (green) and *Tent5a*^*null*^ (purple) mice following 20 min of scrotal exposure at 42 °C. The replicate values of 134 and 123 seminiferous tubule cross sections from *Tent5a*^*WT*^ and *Tent5a*^*null*^ testes, respectively are shown. Student’s *t* test was used to compare each experimental condition to the control condition, ***p < 0.001; ****p < 0.0001; ns, *p* > 0.05.

Given the widespread presence of ATXN2 in SGs across diverse cell types in the male germline, we next investigated if TENT5A plays a role in the germline heat stress response through ATXN2 regulation. We first examined whether testicular *Tent5a* is heat-responsive by subjecting WT mice to scrotal heat, followed by whole testis collection and *Tent5a* quantification. As in somatic cells, *Tent5a* mRNA was upregulated in response to heat (Fig. 5A). Notably, a subtle but significant increase in *Tent5c* mRNA was also observed, suggesting potential overlapping roles for *Tent5a* and *Tent5c* in the germline heat stress response (Fig. 5A). To assess SG formation in *Tent5a* mutants, we examined ATXN2 and G3BP1 in testes from control and *Tent5a*^*null*^ mice after scrotal heat exposure. Resembling the in vitro findings, we observed increased ATXN2-positive granules in *Tent5a*^*null*^ testes, notably in intermediate and B SPG, preleptotene, leptotene, and zygotene SPCs (Fig. 5B). In pachytene SPCs, ATXN2 and G3BP1 largely colocalized in SGs, with *Tent5a*^*null*^ mice showing an increased number of granules (Fig. 5B; panels a, c, e, g, i, k, m, o; arrows). Remarkably, ATXN2-only SG-like structures were observed near stage X pachytene SPCs, and these were also elevated in the *Tent5a-*depleted SPCs (Fig. 5B, panels m, o; arrows). Quantification confirmed a significant increase in G3BP1-positive granules during stages I–III and VII–VIII (Fig. S5A), and a broader upregulation of ATXN2-positive SGs across all seminiferous stages (Fig. S5B). This suggests that increased SG formation in *Tent5a*^*null*^ mice is likely driven by ATXN2. Furthermore, *Tent5a*-deficient germ cells showed reduced colocalization of ATXN2 and G3BP1 in stages X–XII (Fig. 5C). The restricted overlap between the ATXN2 and G3BP1 granules could reflect an altered granule composition. However, the reduced colocalization could also be due to the pronounced decrease of G3BP1 granules in later stages of the seminiferous cycle resulting in a higher proportion of ATXN2-only granules. Indeed, the proportion of ATXN2 signal overlapping with G3BP1 was significantly reduced in *Tent5a*^*null*^ testes during stages X–XII (Fig. S5C), while the fraction of G3BP1 signal overlapping with ATXN2 remained unchanged across the seminiferous epithelial cycle (Fig. S5D). These analyses collectively suggest that ATXN2 is the primary driver of increased heat-induced SGs in the absence of TENT5A.

### TENT5A depletion protects the male germline from heat-induced apoptosis

**Fig. 6:**
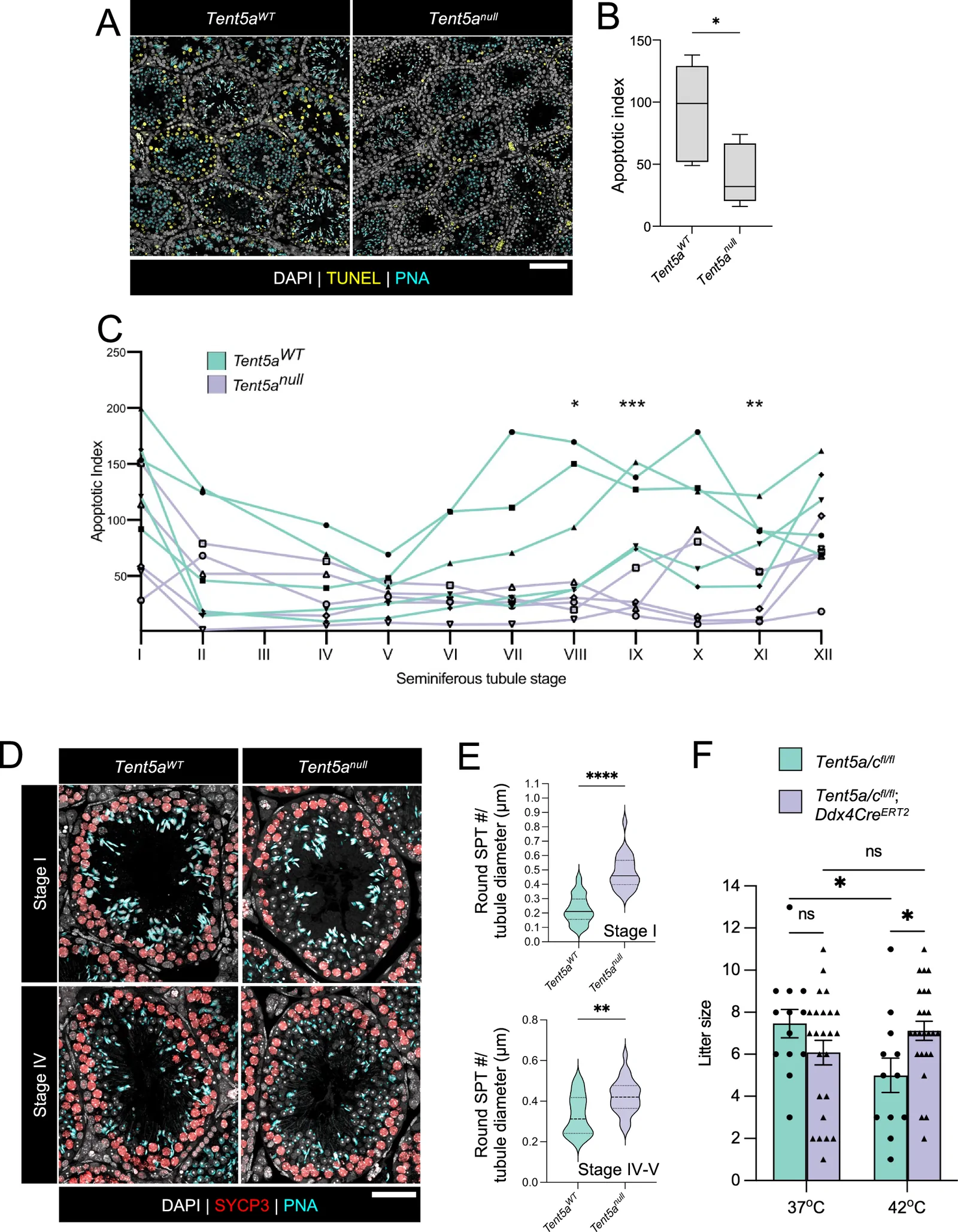
Loss of *Tent5a/*c protects male germ cells against heat exposure. **A** Confocal images showing TUNEL staining (yellow) of testicular cross-sections from *Tent5a*^*WT*^ and *Tent5a*^*null*^ mice 6 h following scrotal exposure to 42 °C. Nuclei and acrosomes were stained with DAPI (white) and PNA (cyan), respectively; Scale bar, 100 µm. **B** Box plot showing the apoptotic index —defined as the number of TUNEL-positive cells per micrometer of tubule diameter – in testicular cross-sections from *Tent5a*^*WT*^ and *Tent5a*^*null*^ mice following scrotal exposure at 42 °C. Boxes represent the interquartile range (25th to 75th percentiles), the horizontal line denotes the median, and whiskers indicate the full data range. The experiments (**A**, **B**) were repeated with 5 control and experimental mouse pairs. **C** Line plot of the apoptotic index across stages I–XII of the seminiferous cycle from five *Tent5a*^*WT*^ and five *Tent5a*^*null*^ mice. **D** Confocal images showing cross sections of stage I and IV seminiferous tubules from WT and *Tent5a*^*null*^ mice one week after scrotal exposure to 42 °C. Nuclei (DAPI, white), acrosomes (PNA, cyan), and spermatocytes (SYCP3, red) are shown. Scale bar, 50 µm. The experiment was repeated with 3 control and experimental mouse pairs. **E**. Violin plots showing the distribution of round spermatid counts —defined as the number of round spermatids per tubule normalized to tubule length (µm) – in stage I (top) and stages IV–V (bottom) seminiferous tubules from *Tent5a*^*WT*^ and *Tent5a*^*null*^ mice one week after scrotal exposure to 42 °C. (Stage I, *Tent5a*^*WT*^, *n* = 41 tubules; Stage I, *Tent5a*^*null*^, *n* = 24 tubules; Stage IV–V, *Tent5a*^*WT*^, *n* = 21 tubules; Stage IV–V, *Tent5a*^*null*^, *n* = 27 tubules). Plots indicate the 1st quartile (bottom), median (middle), and 3rd quartile (top) within the density distribution. **F** Bar chart showing average litter size from tamoxifen-treated *Tent5a*/*c*^*fl/fl*^ and *Tent5a*/*c*^*fl/fl*^; *Ddx4-Cre*^*ERT2*^ mice. Mating outcomes were recorded after four weeks of control (33 °C) or scrotal heat shock (42 °C) treatment. Bar plots represent mean ± SD, with data pooled from 6 (33 °C) and 8 (42 °C) *Tent5a*/*c*^*fl/fl*^ mice, and 10 (33 °C) and 13 (42 °C) *Tent5a/c*^*fl/fl*^; *Ddx4-Cre*^*ERT2*^. Statistical significance was assessed using Student’s *t* test for pairwise comparisons and two-way ANOVA for multiple group comparisons. *p < 0.05; **p < 0.01; ****p < 0.0001.

To assess whether the increase in SGs in the *Tent5a*^*null*^ mice affects cell viability, we next examined apoptosis in testicular sections from control and *Tent5a*^*null*^ mice collected 6 h after scrotal heat exposure. WT mice revealed a stage-dependent trend of cell death as previously described (Figs. 6A–C and S6) [36]. Zygotene SPCs were the most undifferentiated SPCs to undergo heat-induced apoptosis, albeit only at a small rate (Fig. S6A, yellow inset and arrows). Stage I–IV pachytene SPCs exhibited little sensitivity as indicated by the absence of TUNEL staining (Fig. S6B). Few TUNEL-positive cells were detected in stage V SPCs, whereas a moderate number was seen in stage VI SPCs (Fig. S6C, insets and yellow arrows). Stage VII–X pachytene SPCs were the most vulnerable SPCs, exhibiting the most apoptotic cells (Fig. S6D). Finally, the secondary SPCs and stage I–III round spermatids also exhibited moderate apoptosis (Fig. S6A, cyan inset and arrows, S6B). These findings indicate that late pachytene SPCs and early-stage round spermatids are the most sensitive to heat-induced apoptosis consistent with previous observations in cryptorchid animals [37].

Having confirmed that the heat-induced apoptotic trend resembles prior observations, we next compared apoptosis in WT and *Tent5a*^*null*^ mice after heat exposure. Testes from the *Tent5a*^*null*^ mice exhibited markedly fewer TUNEL-positive cells than those of the WT mice (Fig. 6A, B). Seminiferous-stage-wise comparison between the *Tent5a*^*null*^ and *Tent5a*^*WT*^ mice showed similar apoptotic levels during the early seminiferous stages, whereas during the later stages, the *Tent5a*^*null*^ mice exhibited less apoptosis than WT mice (Fig. 6C). In fact, the *Tent5a*^*null*^ mice consistently exhibited reduced apoptosis across all 12 stages of the seminiferous epithelium cycle (Fig. 6C), with the most pronounced differences occurring between stages VI to XII and I (Fig. 6C). This stage-specific reduction in apoptosis indicates a protective role of TENT5A loss against heat-induced germ cell death, particularly during mid to late pachynema.

To assess how the wave of apoptosis impacts the subsequent spermatogenic program, we examined the testis one week after heat exposure. In control mice, spermatogenesis was notably disrupted, marked by a substantial loss of early-stage round spermatids (Fig. 6D). In contrast, this loss was partially rescued in *Tent5a*^*null*^ mice (Fig. 6D). Quantification confirmed a significant increase in round spermatids specifically in stage I–V seminiferous tubules of *Tent5a*^*null*^ mice compared to controls (Fig. 6E). These results suggest that loss of TENT5A mitigates heat-induced damage to spermatogenesis, likely by preserving mid to late-stage pachytene SPCs that subsequently give rise to early-stage round spermatids.

Finally, to evaluate whether the elevated SG numbers and reduced apoptosis and germline loss in *Tent5a*^*null*^ mice translated into physiological outcomes, we conducted post-exposure fertility assays. Given that in addition to *Tent5a*, *Tent5c* is also upregulated in the testis upon heat exposure, albeit at low levels, we speculated that TENT5A and TENT5C might both contribute to the testicular response to elevated temperatures (Fig. 5A). Tent5a and Tent5c are believed to have complementary functions in vivo since *Tent5a* null and *Tent5c* null animals are viable but *Tent5a/c* double nulls rarely survive to weaning [11]. Given that the *Tent5a*^*null*^ mice are subfertile due to non-gametogenic factors [11, 12], and in order to have enough viable animals with *Tent5a/c*-deficient male germ cells, we generated a double inducible germline knockout for *Tent5a* and *Tent5c* (*Tent5a/c*^*fl/fl*^*; Ddx4-Cre*^*ERT2*^) [12, 14, 16]. To avoid sterility caused by complete *Tent5c* deletion, a reduced tamoxifen dose was used to achieve only partial recombination of the *Tent5c* floxed allele. Allele transmission rates were determined by PCR genotyping the offspring from tamoxifen-treated *Tent5a/c*^*fl/fl*^*; Ddx4-Cre*^*ERT2*^ males and wild-type females (Fig. S6E). This confirmed that 96.27% of pups carried the *Tent5a*^*null*^ allele and 42.22% carried the *Tent5c*^*null*^ allele. *Tent5a/c*^*fl/fl*^ and *Tent5a/c*^*fl/fl*^*; Ddx4-Cre*^*ERT2*^ mice were treated with tamoxifen, followed by a five-week recovery period to allow one full cycle of spermatogenesis. Mice were then subjected to scrotal heat and rested for four weeks before fertility evaluation. Without heat exposure, control and *Tent5a/c*^*fl/fl*^*; Ddx4-Cre*^*ERT2*^ mice showed comparable reproductive capacity as determined by litter size (Fig. 6F). As expected, heat-exposed control males exhibited reduced litter size compared to non-heat exposed control males (Fig. 6F). In contrast, the *Tent5a/c*^*fl/fl*^*; Ddx4-Cre*^*ERT2*^ males showed no significant litter size decline after heat exposure (Fig. 6F). The frequency of litters produced did not differ between genotypes or exposure groups, indicating that neither factor changed the mating behavior (Fig. S6F). Hence, the rescued fertility in the *Tent5a/c*^*fl/fl*^*; Ddx4-Cre*^*ERT2*^ males is attributed to better preservation of the germline after heat exposure. These findings collectively support a model in which *Tent5a* germline depletion confers protection against heat-induced germ cell apoptosis, mitigates germ cell loss, and preserves fertility, which is at least in part mediated through the regulation of ATXN2.

## Discussion

Cellular responses to diverse environmental insults converge into a limited set of programs–including activation of the integrated stress and unfolded protein responses, SG formation, and induction of autophagy, senescence or apoptosis [38]. However, the upstream modulators that tailor these programs to specific stressors remain poorly defined. Here, we show that TENT5A, a cytoplasmic poly(A) polymerase, acts as a negative regulator of the heat stress response through dual control of *ATXN2* mRNA and protein. Tight regulation of ATXN2 expression is likely essential, as persistent ATXN2 elevation is associated with many pathological outcomes. These include translation interference [7], oncogenesis [39, 40], as well as chemoresistance across various cancers [41]. Thus, while transient ATXN2 upregulation may promote survival during repairable damage, sustained ATXN2 expression could support tumor progression and therapy resistance.

Elevated testicular temperature is a well-recognized threat to male fertility, and short-term scrotal heating in men or experimental elevation to 42 °C in rodents consistently reduces sperm concentration, motility and DNA integrity with long-lasting effects on pregnancy outcomes [42]. At the cellular level, heat induces oxidative stress [43, 44], disrupts Sertoli cell tight junctions and the blood-testis barrier [45], and activates p53- and BAX-mediated apoptosis in pachytene SPCs and early spermatids [46]. Molecularly, heat activates the unfolded protein response and triggers the integrated stress response via EIF2S1 phosphorylation. This is accompanied by upregulation of the heat shock proteins [47], MAPK cascade and SGs containing DAZL, TIA1 and G3BP1 form selectively in meiotic cells [23]. Despite the progressive understanding of heat stress response in the male germline, the components, regulation and functions of heat-induced SGs remain understudied. Aside from classical stress granule markers, only three studies have reported germline stress granule components in rodents to the best of our knowledge – DAZL, BOULE and SERBP1 [23, 48, 49]. Here, we mapped SG components in the heat-exposed germ cells with stage-specific resolution, identifying ATXN2, DAZL, G3BP1, TIA1 and PABPC1 containing granules. Among them, ATXN2-positive granules were the most prominent and present from intermediate SPG to stage VII/VIII pachytene SPCs. Moreover, given the fertility rescue observed in the *Tent5a/c*^*fl*^*; Ddx4-Cre*^*ERT2*^ conditional knockout mice after exposure, ATXN2-positive granules likely also form in earlier SPGs. Notably, SGs containing either classical markers or ATXN2 were absent in late-pachytene SPCs–a population most prone to heat-induced apoptosis. This link between stage-specific SG absence and heightened apoptotic susceptibility – now extended to include ATXN2-positive granules – reinforces and broadens the DAZL-positive SG pattern previously reported by Kim et al. [23]. Finally, we uncovered a distinct stress granule subtype in leptotene and zygotene SPCs which lacked canonical stress granule markers, indicating a specialized and possibly functionally unique form of SG in these early meiotic cells. Together, these findings highlight the complexity of SGs in the male germline, possibly tailored to meet the distinct physiological needs of each germ cell population.

In germ cells, TENT5Adepletion leads to ATXN2 accumulation, positioning TENT5A as a regulator of SG formation during the hyperthermic stress response. One rodent study identified MAGEB2, a cancer-testis antigen [50], as a germline-specific repressor of SG assembly by reducing G3BP1 translation [27]. While MAGEB2 suppresses heat-induced SG formation, it exerts a similar effect in response to other stressors, including ER and oxidative stress, indicating that it is not a hyperthermia-specific stress responder. Furthermore, whether MAGEB2 expression is induced by heat remains unknown.

The function of heat-induced male germline SGs is another understudied area, with some seemingly contradictory findings. Similar to our current findings, Kim et al. reported that DAZL-containing SGs protect germ cells from heat-induced apoptosis [23]; whereas Lee et al. showed that SGs in MAGEB2 knockout germ cells promoted cell death [27]. Although phenotypically different, it is important to consider that each knockout model alters the composition of SGs in distinct ways, potentially affecting their function and resulting in divergent phenotypes. Supporting this idea, Arimoto and colleagues demonstrated that SGs differing in RACK1 content can drive opposing cell fate outcomes [25]. However, direct evidence for this in the germline context remains lacking. While the composition of germ cell SGs remains largely uncharacterized, comparing SG composition in TENT5A, DAZL, and MAGEB2 knockout germlines could provide valuable insights into how specific SG components influence cell survival versus apoptosis decisions. Thus, SGs should not be viewed as uniformly pro-survival or pro-death structures; rather, their composition and dynamics likely play a key role in modulating the cellular response to heat stress.

Although ATXN2 is a well-established component of somatic SGs, its requirement for SG formation remains debated. An early study identifying ATXN2 as a core SG component also reported that ATXN2 knockdown impaired SG assembly in HEK293 cells treated with 0.5 mM arsenite [7]. In contrast, a recent study found no such requirement in HeLa cells exposed to 0.25 mM arsenite [51]. These discrepancies suggest that ATXN2’s role in SG formation may be influenced by cell type and the intensity of stress. Notably, ATXN2-containing SGs can be induced by various stressors – including heat, oxidative stress (arsenite, selenite, sorbitol, peroxide), and ER stress (DTT, selenite) [7, 52] – yet only heat stress triggered a measurable TENT5A response. This suggests that distinct stress-sensing pathways converge on ATXN2, which then initiates downstream events such as EIF2S1 phosphorylation. Since EIF2S1 can be phosphorylated by different kinases (HRI, PERK, PKR, GCN2), each activated by specific stress signals [53], it would be interesting to investigate whether ATXN2 selectively modulates the activation of these upstream kinases depending on the type of stress.

For decades, it has been known that elevated temperature is detrimental to male germ cell homeostasis, yet the molecular circuitry that senses heat and dictates cell fate remains poorly understood. Our study uncovers a previously unrecognized relay in which TENT5A regulates ATXN2 to fine-tune the cellular heat stress response in somatic and germ cells (Fig. S7). This dynamic is especially critical in the male germline, where we demonstrate stage-specific and cell type-specific SG formation, including previously undescribed SG variants. Importantly, we propose TENT5A to be an upstream regulator and potential master switch of the hyperthermic stress response, integrating both RNA-level and protein–protein control of ATXN2. Given ATXN2’s association with apoptosis modulation and oncogenesis, understanding this regulatory axis has implications not only for fertility preservation under heat stress, but also for broader pathophysiological contexts. Together, our findings advance the current understanding of SG biology, highlight the specialized features of the germline stress response, and reveal TENT5A as a key node linking temperature sensing to cell fate decisions.

## Experimental model

### In vivo animal studies

All animal procedures were conducted in accordance with the NIH Guide for the Care and Use of Laboratory Animals and were approved by the National Institute of Environmental Health Sciences (NIEHS) Institutional Animal Care and Use Committee (IACUC; ASP protocol 2019-0004).

#### Mouse strains

Mouse strains used in this study were: loxP-flanked *Tent5a, Tent5a*^*fl*^ (B6.129S-Tent5a<tm1Mmo > ); *CMV-Cre* (B6.C-Tg(CMV-cre)1Cgn/J, The Jackson Laboratory, IMSR_JAX:006054) for global conversion of floxed alleles [54]; *Ddx4-Cre*^*ERT2*^ (FVB/N-Tg(Ddx4-cre/ERT2)1Dcas/J, The Jackson Laboratory, IMSR_JAX:024760) [55] for germline conversion of the loxP-flanked *Tent5c*, *Tent5c*^*fl*^ (B6.Cg-Tent5c<tm1Mmo > ) [16] and *Tent5a*^*fl*^ alleles upon tamoxifen administration. All mice were housed in the NIEHS animal facility under controlled conditions: 12-h light/dark cycles, 50% ± 15% humidity, and ambient temperatures of 20.5–23.9 °C, with ad libitum access to food and water. Mouse genotyping was performed by Transnetyx (Memphis, TN, USA). Automated DNA extraction from ear punches was followed by real-time PCR for routine colony maintenance and identification of experimental and control animals. Adult post-pubertal male mice were used for the study. Males were used for the in vivo analyses due to the hypersensitivity of the testis to heat. Littermates were assigned to the different experimental groups according to their genotype.

#### Generation of the Tent5a^fl^ allele

The *Tent5a*^*fl*^ allele was generated by CRISPR/Cas9-mediated genome editing in G4 (B6129F1) embryonic stem (ES) cells. Two loxP sites were inserted to flank a 691 bp region that includes 80 bp of intron 1–2, the entirety of exon 2 (which encodes approximately 41% of the TENT5A protein), and 64 bp of the 3′ untranslated region (UTR). A single Cas9-induced double-stranded break was targeted to the intronic sequence ATGGGCGTCACGATCGGGGANGG using the PX459v2 sgRNA/Cas9/Puro delivery plasmid (Addgene #62988), a gift from Feng Zhang [56]. A single circular repair template was used to introduce both loxP sites, consisting of the following: 5’ homology arm (1355 bp, chr9:85,326,907-85,328,261 GRCm38/mm10), the 5’ loxP with SphI restriction site, the internal homology arm (691 bp, chr9:85,326,195-85,326,885 GRCm38/mm10) including exon 2, 3’ loxP with NdeI restriction site, and the 3’ homology arm (508 bp, chr9:85,325,626-85,326,133 GRCm38/mm10). The unique restriction sites included with each loxP site were to facilitate screening. ES cell clones were screened by high-fidelity PCR using a single 3 kb amplicon spanning both homology arms, followed by restriction digest. The correct insertion of loxP sites and the integrity of exon 2 were confirmed by direct sequencing. In silico analysis indicated that the Cas9 target site had no predicted off-target effects within a 25 Mbp window on chromosome 9; therefore, off-target screening was not performed. Correctly targeted homozygous ES clones were microinjected into wild-type blastocysts for chimera generation. Germline-transmitting male chimeras were bred to C57BL/6J wild-type females, and the *Tent5a*^*fl*^ allele was confirmed by resequencing in the F1 offspring. The resulting line was maintained and expanded on a C57BL/6J background, and genotyping of the *Tent5a*^*fl*^ colony was performed using allele-specific primer/probe assays by Transnetyx (Cordova, TN, USA).

#### Generation of experimental mice

Constitutive Tent5a knockout mice (*Tent5a*^*null*^) were generated by crossing *Tent5a*^*fl/WT*^ mice with CMV-Cre transgenic mice (B6.C-Tg(CMV-cre)1Cgn/J) to obtain *Tent5a*^*null/WT*^ offspring. These heterozygous animals were then intercrossed to generate wild-type (control, *Tent5a*^*WT/WT*^) and homozygous knockout (experimental, *Tent5a*^*null/null*^, hereafter referred to as *Tent5a*^*null*^) mice. The germline conditional *Tent5a/c* knockout mice (*Tent5a/c*^*fl*^*; Ddx4-Cre*^*ERT2*^) were generated by first crossing *Tent5a*^*fl/WT*^ and *Tent5c*^*fl/WT*^ mice to obtain *Tent5a*^*fl/WT*^*; Tent5c*^*fl/WT*^ double heterozygotes. These were subsequently bred with *Ddx4-Cre*^*ERT2*^ transgenic mice until homozygosity for floxed alleles was achieved in the presence of the Cre recombinase transgene. Tamoxifen-induced recombination was achieved by administering 75 mg/kg tamoxifen via intraperitoneal injection for five consecutive days. Control mice consisted of tamoxifen-injected *Tent5a*^*fl/fl*^*; Tent5c*^*fl/fl*^ mice lacking the *Ddx4-Cre*^*ERT2*^ transgene.

### Cell lines and primary culture

#### Cell lines

The 17Cl1 cell line (derived from 3T3 cells; BEI Resources, Cat. No. NR-53719) was maintained in Dulbecco’s Modified Eagle Medium (DMEM; Life Technologies) supplemented with 10% fetal bovine serum (FBS; Gemini Bio-Products), 4 mM L-glutamine (Invitrogen), and 1 mM sodium pyruvate (Invitrogen). HEK293T (HEK293T/17, CRL-11268™) and HeLa (CCL-2) cells were obtained from the American Type Culture Collection and maintained in DMEM supplemented with 10% FBS and 4 mM L-glutamine. MEF cells were isolated from E13.5 embryos (see section “Mouse embryonic fibroblast (MEF) cell isolation”) and cultured in DMEM containing 10% FBS and 2 mM L-glutamine. All cells were maintained at 37 °C in an atmosphere of 20% oxygen and 5% carbon dioxide, and were routinely tested for mycoplasma contamination.

#### Mouse embryonic fibroblast (MEF) isolation

MEF cells were isolated following a protocol adapted from [57]. *Tent5a*^*null/WT*^ males were housed with *Tent5a*^*null/WT*^ females, and the morning of vaginal plug detection was designated embryonic day 0.5 (E0.5). At E13.5, pregnant females were euthanized, and the uterine horns were removed and placed in PBS. Embryos were carefully dissected from the uterine horns and transferred to fresh PBS. After detachment from the placenta and a gentle PBS rinse, embryos were decapitated, and internal organs were removed. The remaining embryonic bodies were minced in tissue culture-grade 0.25% trypsin using sterile razor blades until finely fragmented, followed by incubation at 37 °C for 10 min. The suspension was mixed thoroughly by pipetting and incubated for an additional 5 min at 37 °C. Trypsin was neutralized with MEF culture medium, and the suspension was filtered through a 40-μm strainer into a 15-mL conical tube. Cells were collected by centrifugation at 500 × *g* for 5 min at 4 °C and resuspended in MEF culture medium (DMEM supplemented with 10% FBS, 2 mM L-glutamine, and 1× penicillin/streptomycin). MEFs were maintained under standard tissue culture conditions.

#### MEF cell line immortalization

Isolated mouse embryonic fibroblast (MEF) cell lines were immortalized by constitutive expression of the Simian Vacuolating Virus 40 large T antigen (SV40 Large T), delivered via recombinant lentiviruses. Lentivirus production was performed using the transfer vector Ef1a_Large T-antigen_IRES_Puro (Addgene plasmid #18922), following published protocols [58]. Cells were transduced twice at a multiplicity of infection (MOI) of 5 and subsequently cultured in puromycin-containing selection medium for two weeks prior to the generation of clonal cell lines.

## Methods details

### Cellular stress conditions

To induce cellular stress, 17Cl1 and HEK293 cells were treated with 1 mM H₂O₂ (Fisher Scientific, BP2633-500), 2 mM dithiothreitol (DTT), or 400 μM sodium arsenite, or exposed to 42 °C or 4 °C for 1–1.5 h. Subsequent heat stress experiments were performed at 42 °C or 44 °C for 1–6 h in 17Cl1 and MEF cells, and 1–2 h in HeLa and HEK293 cells. Heating times were determined to capture the cells that were heat-stressed but not yet detached.

### RNA extraction

Frozen testes or cultured cells were homogenized in TRIzol reagent (Invitrogen) and brought to room temperature. Chloroform (200 μL per 1 mL of TRIzol) was added, and samples were tightly capped, shaken vigorously for 15 s, and incubated at room temperature for 2 min. Phase separation was performed by centrifugation at 12,000 *g* for 15 min at 4 °C. The aqueous phase was transferred to fresh tubes, and RNA was precipitated by adding isopropanol (500 μL per 1 mL of TRIzol), followed by a 10-min incubation at room temperature. Samples were centrifuged at 12,000 *g* for 10 min at 4 °C, and RNA pellets were washed in 75% ethanol (1 mL per 1 mL of TRIzol used) with brief vortexing. Pellets were then centrifuged at 7500 *g* for 5 min at 4 °C, air-dried briefly, and resuspended in an appropriate volume of buffer. Alternatively, RNA was extracted using the RNeasy Kit (Qiagen, Cat. No. 74104) according to the manufacturer’s instructions. RNA concentration was measured using a NanoDrop 2000 spectrophotometer (Thermo Scientific). For gene expression analysis, 1 μg of RNA was treated with 10^−5^ U of DNase I (Invitrogen, 18068-015) per μg of RNA for 15 min at room temperature. DNase I was inactivated by adding EDTA to a final concentration of 2.5 mM, followed by incubation at 65 °C for 10 min.

### cDNA synthesis

DNase I-treated RNA was mixed with random hexamers, dNTPs, First-Strand Buffer, DTT, RNase inhibitor, and SuperScript II Reverse Transcriptase (Invitrogen, Cat. No. 10004925) to final concentrations of 2.5 μM hexamers, 500 μM dNTPs, 1× buffer, 10 mM DTT, 2 U/μL RNase inhibitor, and 2.5 U/μL SuperScript II. The reaction mixture was incubated at room temperature for 10 min, followed by 42 °C for 50 min, and then 75 °C for 15 min to inactivate the enzyme. The resulting cDNA was diluted 1:5 with nuclease-free water for downstream applications.

### Quantitative PCR (qPCR)

Real-time qPCR was performed using the SsoAdvanced Universal SYBR Green Supermix (Bio-Rad, Cat. No. 1725272) according to the manufacturer’s instructions. Reactions were run on the CFX Duet Real-Time PCR System (Bio-Rad). Primer sequences are listed in Table S2.

### Immunofluorescence for cultured cells

Cells were seeded on 22-mm × 22-mm coverslips sterilized with 70% ethanol. After exposure to the stress conditions described above, cells were fixed in 4% paraformaldehyde for 10 min at room temperature, followed by three washes in PBS. Cells were then permeabilized with 0.5% Triton X-100 in PBS for 5 min and washed with PBS-T (0.2% Tween-20) for 5 min at room temperature. Blocking was performed using CytoVista™ Blocking Buffer (ThermoFisher Scientific, V11307) for 1 h at room temperature. Cells were incubated with primary antibodies diluted in the same buffer for 1 h at 37 °C or overnight at 4 °C (see Table S2 for antibody details). After three PBS-T washes, cells were incubated with the appropriate secondary antibody (diluted in CytoVista™ Blocking Buffer) for 1 h at room temperature, followed by three final PBS-T washes. Coverslips were mounted onto Fisherbrand™ Superfrost™ Plus microscope slides (Fisher Scientific, Cat. No. 12-550-15) using VECTASHIELD® Vibrance™ Antifade Mounting Medium with DAPI.

### Polymerase chain reaction (PCR)

PCR reactions were performed using either REDTaq ReadyMix™ PCR Reaction Mix with MgCl₂ (Sigma-Aldrich, Cat. No. R2523) or LongAmp Taq 2X Master Mix (New England Biolabs, Cat. No. M0287), according to the manufacturers’ protocols. For REDTaq reactions, template DNA and forward and reverse primers (final concentration of 1 μM each) were mixed with REDTaq ReadyMix™ and nuclease-free water to a final reaction volume equal to 2X the volume of ReadyMix used. Thermal cycling conditions were as follows: initial denaturation at 95 °C for 3 min; 25–35 cycles of 95 °C for 30 s (denaturation), primer-specific annealing temperature for 30 s (annealing), and 72 °C for 30 s (elongation); followed by a final extension at 72 °C for 5 min. For LongAmp Taq reactions, primers were used at a final concentration of 0.4 μM. Template DNA and water were added to a final reaction volume corresponding to a 1X Master Mix concentration. Cycling conditions included an initial denaturation at 94 °C for 30 s; 25–35 cycles of 94 °C for 10–30 s (denaturation), annealing temperature for 15–60 s (annealing), and 65 °C for 50 s per kilobase of expected product size (elongation). Annealing temperatures and cycle numbers were empirically optimized for each primer set. PCR products were analyzed on 0.75–2.5% agarose gels, depending on the size of the expected amplicon. All primer sequences are listed in Table S2.

### Transfection

HEK293 cells were transfected with FHpHG or pcDNA expression constructs using Lipofectamine™ 3000 Transfection Reagent (ThermoFisher Scientific, Cat. No. L3000001) according to the manufacturer’s instructions. Briefly, the appropriate amounts of DNA and Lipofectamine 3000 were each diluted in Opti-MEM™ Reduced Serum Medium (Gibco, Cat. No. 31985070). The P3000™ reagent (provided in the kit) was added to the DNA dilution. After brief vortexing, the diluted DNA and transfection reagent were combined and incubated at room temperature for 15 min before being added dropwise to the cell culture medium.

### Cell viability assay

HEK293 and MEF cells were seeded in 96-well plates at densities of 25,000 and 12,500 cells per well, respectively. HEK293 cells were transfected with the appropriate expression constructs 16 h after seeding and incubated for an additional 48 h. HEK293 and MEF cells were then subjected to heat shock for 16 and 6 h, respectively, after which viability was assessed using the CellTiter-Glo® 2.0 Cell Viability Assay (Promega, Cat. No. G9242) according to the manufacturer’s instructions. Viability was calculated relative to non-heat-shocked controls.

### Protein extraction and quantification

Cells or tissues were homogenized in RIPA Lysis and Extraction Buffer (ThermoFisher Scientific, Cat. No. 89900) supplemented with 1X Halt™ Protease and Phosphatase Inhibitor Cocktail (ThermoFisher Scientific, Cat. No. 78442) using either a bead mill or a sonicator. Lysates were incubated on ice for at least 5 min, then centrifuged at 21,000 *g* for 15 min at 4 °C. The resulting supernatant, containing the soluble protein fraction, was transferred to fresh tubes. Protein concentration was determined using the Pierce™ BCA Protein Assay Kit (ThermoFisher Scientific, Cat. No. 23225) according to the manufacturer’s instructions.

### Western blot

Heat-denatured protein samples were separated on 10% or 4–20% Criterion™ Tris-HCl precast gels (Bio-Rad) and transferred onto nitrocellulose membranes (Invitrogen, IB301001). Membranes were blocked in 5% (w/v) non-fat milk prepared in 1X TBS-T for 1 h at room temperature. Primary antibodies (listed in Table S2) were applied and incubated overnight at 4 °C. After three washes with 1X TBS-T, membranes were incubated with the appropriate secondary antibody for 1 h at room temperature. Western blots were repeated at least three times for each experiment. Protein bands were visualized using the Clarity™ Western ECL Substrate (Bio-Rad, Cat. No. 1705060), and signals were captured using the Amersham ImageQuant 800 system. Uncropped images are provided as supplementary information.

### Production of shRNA lentiviral vectors

Mouse pGIPZ lentiviral shRNA vectors (ThermoFisher Scientific) were obtained from the NIEHS Epigenetics Core Facility and originally purchased from Horizon. Sequences for shRNAs targeting *Tent5a* (shTent5a), *Tent5b* (shTent5b), and the non-targeting control (shCTL) are listed in Table S2. Lentiviral particles were produced in HEK293 cells following established protocols [59]. Cells were transiently transfected using Lipofectamine™ 2000 with pGIPZ vectors encoding shCTL, shTent5a, or shTent5b, along with the packaging plasmids psPAX2 (Addgene #12260) and pMD2.G (Addgene #12259), both a generous gift from Dr. Didier Trono. After 48 h, the supernatant containing lentiviral particles was collected, aliquoted, and stored at –80 °C. Lentiviral titers were quantified by digital droplet PCR (ddPCR) and reported as the number of integrated viral particles per genome in transduced HEK293T cells.

### Generation of 17Cl1 shCTL and shTENT5AB stable cells

Stable 17Cl1 cell lines expressing shCTL or shTENT5AB were generated using an shRNA-containing lentiviral knockdown system. Briefly, 20,000 cells were seeded per well in 6-well plates. The following morning, cells were infected with lentiviruses encoding shCTL (MOI 20), shTent5a (MOI 10), and shTent5b (MOI 10). After 24 h, the viral supernatant was replaced with fresh medium containing 2 µg/mL puromycin. Following a 48-h selection period, cells were maintained in 1 µg/mL puromycin to establish stable knockdown lines. Knockdown efficiency of *Tent5a* and *Tent5b* in the shTENT5AB stable cells was evaluated by qPCR.

### Poly(A) tail purification

Poly(A)-tailed mRNAs were enriched from total RNA using Dynabeads™ Oligo(dT)25 beads (ThermoFisher Scientific, Cat. No. 61002). Briefly, 20 μL of beads per sample were transferred to low-binding tubes, and the storage buffer was removed using a magnetic separation rack (MSR). Beads were washed once with 40 μL of binding buffer (20 mM Tris-HCl, pH 7.5; 1.0 M LiCl; 2 mM EDTA) and resuspended in 40 μL of the same buffer. A total of 5 μg of cellular RNA from shCTL or shTent5a/b cell lines was diluted with nuclease-free water to a final volume of 40 μL, then mixed with 40 μL of binding buffer. The RNA was denatured at 65 °C for 2 min, combined with 40 μL of pre-washed oligo(dT) beads, and incubated at room temperature for 5 min. Beads were separated using the MSR, and washed twice with wash buffer (10 mM Tris-HCl, pH 7.5; 0.15 M LiCl; 1 mM EDTA). For elution, beads were resuspended in 10 μL of elution buffer (10 mM Tris-HCl, pH 7.5) and incubated at 80 °C for 2 min. Poly(A)-enriched RNA was immediately separated from the beads using the MSR.

### Direct RNA-sequencing library preparation

Direct RNA sequencing (DRS) libraries were prepared using the Direct RNA Sequencing Kit (SQK-RNA002, Oxford Nanopore Technologies) according to the manufacturer’s instructions, with minor modifications [60, 61]. Briefly, 8 μL of poly(A)-enriched RNA was mixed with 3 μL of ligation buffer (6% PEG8000 in 10X T4 DNA ligation buffer; NEB, B0202S), 1.5 μL of T4 DNA ligase (NEB, M0202L), 1.5 μL of a 100 nM barcoded-oligonucleotide duplex mix, and 1 μL of a 0.1 ng/μL RNA standard mix [60]. The ligation reaction was incubated at room temperature for 60 min. Barcode-ligated RNA was then converted to an RNA/cDNA hybrid by reverse transcription. For reverse transcription, samples were mixed with 9 μL of nuclease-free water, 2 μL of 10 mM dNTPs, 8 μL of 5× First-Strand Buffer, 4 μL of 100 mM DTT, and 2 μL of SuperScript II reverse transcriptase (ThermoFisher, 18064022), followed by incubation at 50 °C for 50 min, 70 °C for 10 min, and cooling to 4 °C in a thermocycler. RNA/cDNA hybrids were purified using 72 μL of RNAClean XP beads (Beckman Coulter, A63987) with a 5-min incubation at room temperature. Beads were washed with 150 μL of 70% ethanol, resuspended in 20 μL of nuclease-free water, and incubated for 5 min at room temperature to elute the RNA/cDNA hybrids. For adapter ligation, 8 μL of ligation buffer (6% PEG in 10× T4 DNA ligation buffer), 6 μL of RMX-RNA adapter (from the SQK-RNA002 kit), 3 μL of nuclease-free water, and 3 μL of T4 DNA ligase were added to the eluted sample in a final 40 μL reaction. The mixture was incubated at room temperature for 15 min. Adapter-ligated RNA/cDNA hybrids were purified using 40 μL of RNAClean XP beads, incubated for 5 min at room temperature, and washed twice with 150 μL of wash buffer (WSB, supplied with the kit). Beads were resuspended in 21 μL of elution buffer (EB, SQK-RNA002 kit) and incubated for 15 min at room temperature. Final libraries were eluted using an MSR. Libraries were prepared for sequencing on an R9.4.1 flow cell using the ONT Flow Cell Priming Kit (EXP-FLP002) per the manufacturer’s instructions. Flow cell quality was first assessed using a MinION instrument. For priming, 800 μL of FLT/FB mix (30 μL FLT in 1 mL FB) was loaded through the priming port and incubated for 5 min. During this time, the library was prepared by mixing 37.5 μL RNA Running Buffer (RRB, SQK-RNA002) and 17.5 μL of nuclease-free water with the eluted library. After priming, an additional 200 μL of FLT/FB mix was added, and the library was loaded through the spot-on port. Sequencing was carried out on an ONT MinION instrument, and Fast5 files were collected using ONT MinKNOW software (v5.2.15).

### Data analysis and poly(A) tail length determination

Direct RNA sequencing data were basecalled using Guppy (v5.0.10, Oxford Nanopore Technologies) and demultiplexed with DeePlexiCon [62]. Demultiplexed reads were aligned to the mouse genome (mm10), annotated with GENCODE vM17, using minimap2 (v2.20) [63], and the resulting alignments were indexed with SAMtools [64]. Poly(A) tail length quantification was performed using the Nanopolish pipeline [65, 66]. Reads were filtered to retain only those with a poly(A) tail quality score qc_tag = “PASS” and a confidence interval greater than 0.95.

### Generation of the PGL3-luciferase-*Atxn2* 3’UTR construct and luciferase assay

The pGL3-Control vector (Promega; discontinued) was a kind gift from Dr. Tatsuya Sueyoshi. The *Atxn2* 3′UTR sequence was amplified by PCR using primers containing XbaI restriction sites (Table S2). The pGL3-Control vector was linearized by XbaI digestion, and the amplified *Atxn2* 3′UTR fragment was ligated into the vector backbone. Correct insertion was confirmed by Sanger sequencing (Azenta). HEK293 cells were seeded at 150,000 cells per well in 24-well plates and transfected 16 h later with the *Atxn2* 3′UTR-Luc construct, the pRL-TK-Renilla luciferase plasmid (Promega, E224A), and either the FHpHG vector or FHpHG-*Tent5a* expression construct at final concentrations of 50 ng/well, 10 ng/well, and 200 ng/well, respectively [67]. After 48 h, luciferase activity was measured using the Dual-Luciferase® Reporter Assay System (Promega, E1910), according to the manufacturer’s instructions. Firefly luciferase activity was normalized to Renilla luciferase activity from the pRL-TK plasmid, and fold changes were calculated relative to cells transfected with the control 3′UTR.

### Co-immunoprecipitation

HEK293 cells were seeded at a density of 3 × 10⁶ cells per 10-cm dish and transfected 16 h later with 10 μg of FHpHG constructs. After 48 h, cells were lysed under native (non-denaturing) conditions in 1 mL Pierce™ IP Lysis Buffer (ThermoFisher Scientific, Cat. No. 87788) supplemented with a protease and phosphatase inhibitor cocktail. RNase inhibitors were not included, and no exogenous RNase treatment was performed. Lysates were passed through a 25 G needle five times and incubated on ice for 15 min. The soluble fraction was collected by centrifugation at 15,000 *g* for 15 minutes at 4 °C, and supernatants were transferred to fresh tubes. A small aliquot was reserved as input, and the remaining lysate was incubated with GFP nanobody for 3 hours at 4 °C with rotation [68]. The GFP nanobody-bound TENT5A and its interacting proteins were washed five times with HIP buffer (50 mM Tris-HCl, 150 mM NaCl, 1% Triton X-100), then eluted by boiling in SDS sample buffer for 5 minutes. Input and immunoprecipitated samples were analyzed by Western blotting.

### Scrotal heat exposure

To study the role of *Tent5a* in the male germline, adult male mice were subjected to scrotal heat exposure. A custom 3D-printed platform was used to position the mice such that their lower extremities were submerged in a water bath maintained at either 33 °C (control) or 42 °C (heat-exposed). Prior to heat exposure, temperature transponders were implanted subcutaneously in the dorsal region to enable monitoring of core body temperature throughout the procedure. Core body temperature was recorded at 2-min intervals using a temperature-sensing radio frequency identification reader. While no active temperature regulation was applied, 42.4 C was specified as the upper temperature threshold to stop the procedure [69]. No mice fell outside the specified range during any experiment. Mice were anesthetized using either isoflurane inhalation or an intraperitoneal injection of ketamine:xylazine (100 mg/kg : 7 mg/kg body weight). Depending on the experimental endpoint, mice were either euthanized immediately after the procedure or allowed to recover under observation. For TUNEL assays, mice were euthanized 6 h post-exposure.

### Tissue processing for immunofluorescence (IF)

Following the appropriate treatments, mice were euthanized, and testes were collected and washed in PBS. Tissues were fixed in 4% paraformaldehyde with gentle shaking for 24–48 h at 4 °C. After fixation, testes were rinsed three times in PBS and transferred to 70% ethanol for at least 48 h. Subsequently, tissues were dehydrated, embedded in paraffin, and sectioned at a thickness of 5 μm. Paraffin sections were deparaffinized by three serial incubations in CitriSolv clearing reagent (ThermoFisher Scientific, Cat. No. 22-143-975), then rehydrated through a graded ethanol series.

### Immunofluorescence for testicular sections

Following rehydration, antigen retrieval was performed using VECTOR Laboratories Antigen Unmasking Solution (H-3300) according to the manufacturer’s instructions. Sections were then blocked in a solution containing 0.4% (v/v) Triton X-100, 1% BSA, and 5% normal goat serum for 60 min at room temperature. Tissues were incubated overnight at 4 °C with primary antibodies diluted in 0.4% Triton X-100 in PBS. After washing in PBS, sections were incubated with fluorophore-conjugated secondary antibodies diluted in the same buffer for 90 min at room temperature. Finally, slides were washed three times in PBS and mounted using DAPI-containing mounting medium (Vector Laboratories, H-1800).

### Confocal imaging

All immunofluorescence images were acquired using a Zeiss LSM 980 confocal microscope. Laser power and gain settings were kept constant for comparative imaging. Image processing was performed using Zeiss Zen Blue software. Quantification of stress granules (SGs) in cultured cells and testes, as well as analysis of apoptosis and germ cell loss, was conducted using FIJI/ImageJ [70]. Thresholds for SG detection were empirically determined prior to full analysis using a subset of representative images. Colocalization of G3BP1- and ATXN2-positive SGs was analyzed using the JaCoP plugin for FIJI [71].

### TUNEL assay

Post-heat-shock apoptotic germ cells were detected by terminal deoxynucleotidyl transferase dUTP nick end labeling (TUNEL) using the In Situ Cell Death Detection Kit, Fluorescein (Millipore Sigma, Cat. No. 11684795910), according to the manufacturer’s instructions. Briefly, 5 μm testicular sections from formalin-fixed, paraffin-embedded tissues were deparaffinized and rehydrated (see Materials and Methods section: “Tissue processing for immunofluorescence”). Antigen retrieval was performed by boiling sections in citrate-based Antigen Unmasking Solution (Vector Laboratories, H-3300) using a microwave, followed by permeabilization in 0.4% Triton X-100. Sections were incubated with TUNEL labeling solution prepared by diluting the fluorescein-conjugated enzyme solution 1:10 with fluorescein labeling buffer (both provided in the kit) and incubated at 37 °C for 1 h. After three PBS washes, sections were mounted using DAPI-containing mounting medium. Fluorescein signal from apoptotic cells was detected with excitation at 488 nm and emission collected between 491 and 525 nm.

### Fertility assay

For the fertility study, tamoxifen-treated *Tent5a/c*^*fl/fl*^ and *Tent5a/c*^*fl/fl*^*; Ddx4-Cre*^*ERT2*^ males were housed with 6–8-week-old virgin C57BL/6J females beginning four weeks after scrotal heat exposure.

## Quantification and statistical analysis

The center and dispersion values, statistical tests, and number of variables used can be found in the figure legends. Results were considered not statistically significant if the *p* values exceeded 0.05. The *p* value ranges are defined in each figure legend and shown in the figures. Sample sizes were chosen based on the expected effect size and variance typically observed for each type of assay. For in vivo studies, sample sizes were estimated based on previous work on the effect of DAZL on scrotal heat exposure, which used similar exposure protocols and measured equivalent outcomes [23]. For studies using wild-type mice, littermates were randomized to the control or treated groups. For studies using genetically modified mice, animals were first grouped by genotype and then randomly assigned to each experimental group. Image acquisition was performed in a blinded manner with respect to genotype and treatment. Quantification of SGs and apoptosis scoring were conducted using standardized parameters applied uniformly across samples to minimize operator-dependent bias.

## Supplementary information

Supplementary information

Table S1

Table S2

Uncropped Western blots

## Data Availability

The datasets for the analysis of transcripts’ poly(A) tails have been deposited under GEO accession number GSE311487.
