# Supplementary material for: The TENT5A-ATXN2 axis modulates germline and somatic cell survival during heat stress
Source: Cell Death Dis. 2026 Jul 14;17(1):871. doi: 10.1038/s41419-026-09086-1 (PMC13646263; doi:10.1038/s41419-026-09086-1)
Supplement: Supplementary file 1 [file 41419_2026_9086_MOESM1_ESM.pdf]

Inventory of supplementary material

The TENT5A-ATXN2 axis modulates germline and somatic cell survival during heat stress.

Ru-Pin Alicia Chi[1], Ankit Gupta[1], Yin Li[1], Marine Baptissart[1], Dongwon Lee[1], Brian N. Papas[2] and Marcos Morgan[1,3]

Supplementary figures

Figure S1. Different exposures induce the formation of stress granules and ER stress.

Figure S2. Validation of TENT5A overexpression and knockout in HEK293 and MEF cells, respectively.

Figure S3. ATXN2 localizes to G3BP1-positive granules.

Figure S4. PABPC1, TIA1, and DAZL form stress granules in early and mid-pachytene SPCs.

Figure S5. Quantitative analysis of ATXN2 and G3BP1-positive granules through different stages of the seminiferous cycle.

Figure S6. Loss of *Tent5a* confers protective effects against heat exposure.

Figure S7. TENT5A-mediated downregulation of ATXN2-positive stress granules during heat stress promotes apoptosis.

Supplementary tables

Table S1. Expression levels of the different *Tent5* subfamily members in 17Cl1, HEK293, HeLa, and MEF cells.

Table S2. Summary of oligos, shRNAs, and antibodies used.

Original uncropped Western blots

Academic journals reporting checklist

Supplementary figures

Figure S1

A

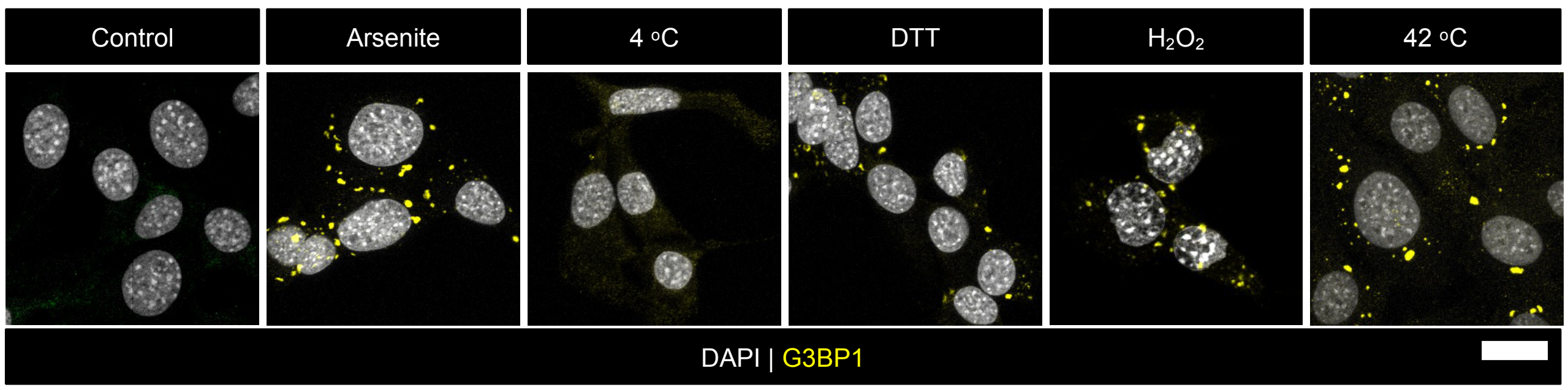

B

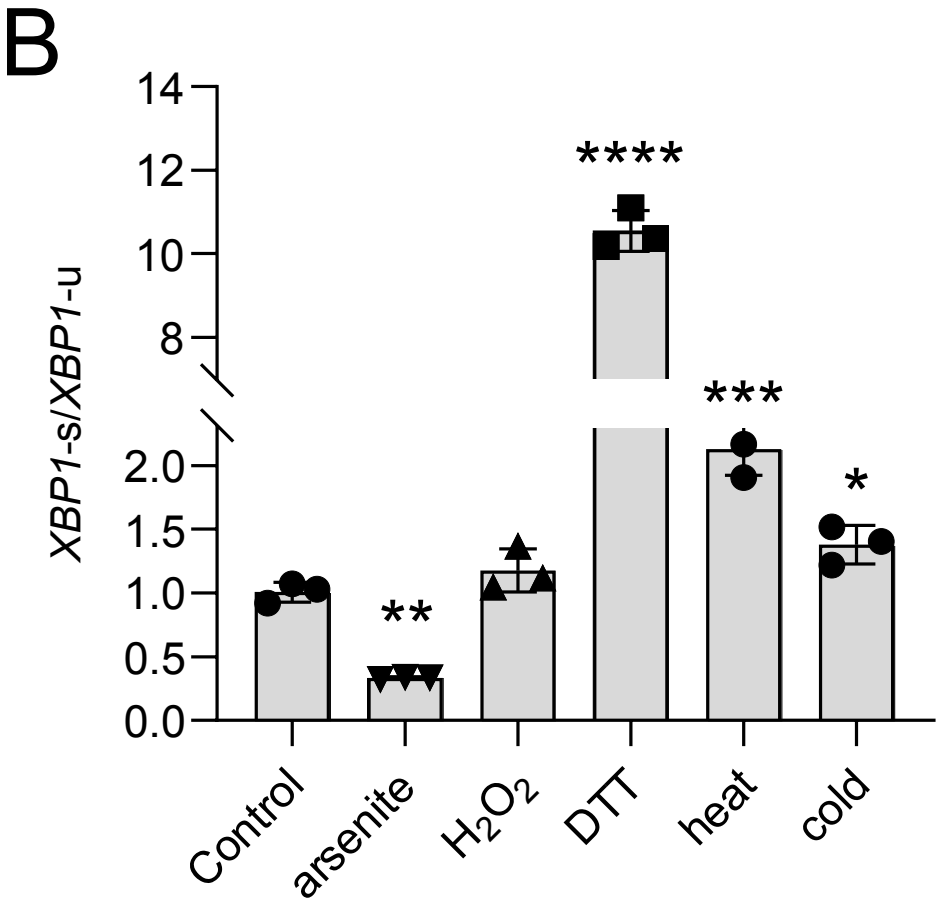

Figure S1: Different exposures induce the formation of stress granules and ER stress.

A. Confocal scan showing G3BP1 (yellow) and DAPI (white) in 17Cl1 cells exposed to sodium arsenite, 4 °C, DTT, H2O2, and 42 °C. Scale bar = 20 *μ*m. The experiment was repeated twice.

B. Bar plot of the ratio of spliced *XBP1* (*XBP1-s*) to unspliced *XBP1* (*XBP1-u*) in HEK293 cells exposed to sodium arsenite, H2O2, DTT, heat, and cold, as determined by qPCR. Results are presented as mean ± SD from three biological replicates. Statistical significance was determined by two-tailed Student's t-test: *, $p < 0.05$; **, $p < 0.01$; ***, $p < 0.001$; ****, $p < 0.0001$.

Figure S2

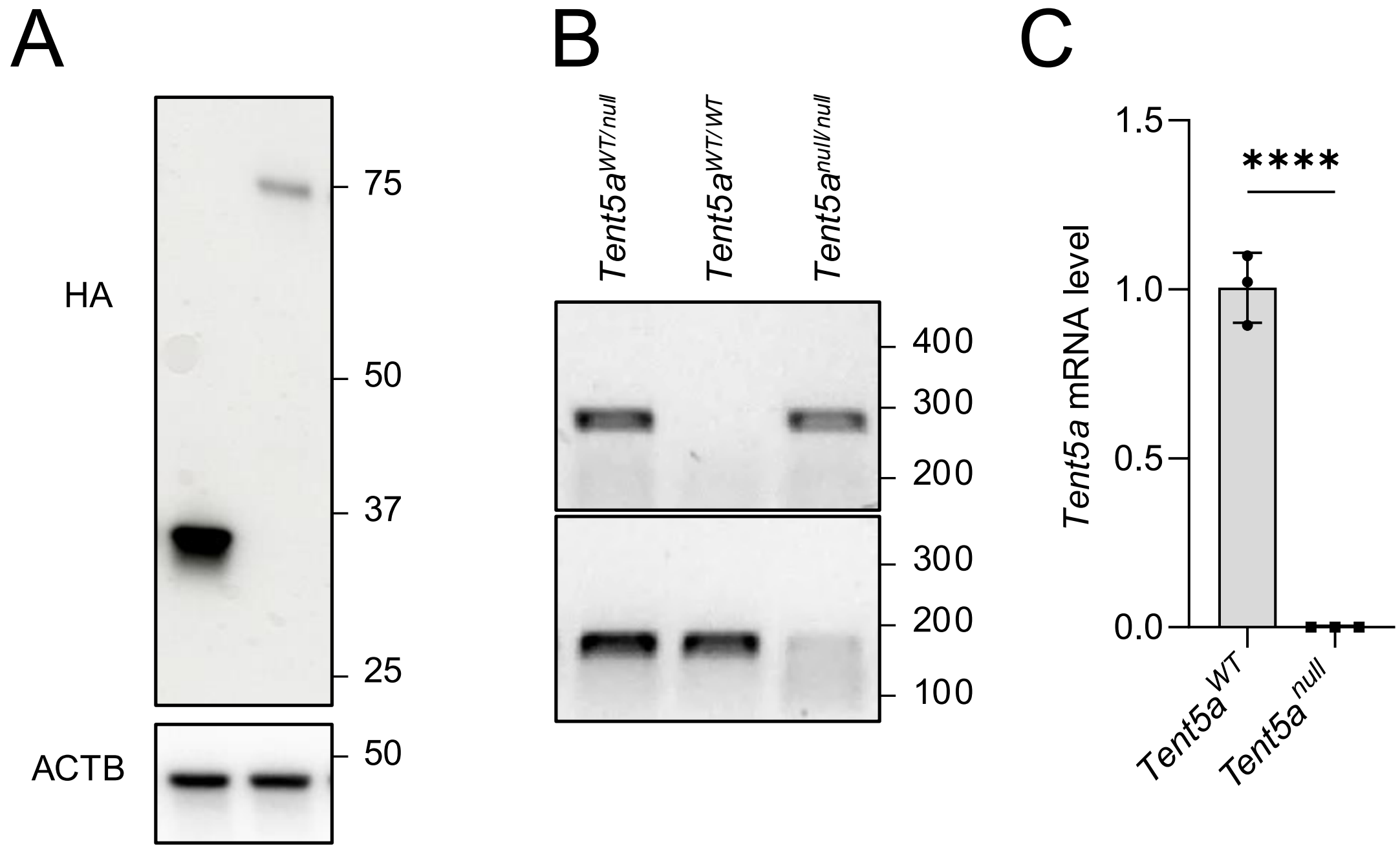

Figure S2. Validation of TENT5A overexpression and knockout in HEK293 and MEF cells, respectively.

A. Western blot of extracts from HEK293 cells transfected with FHpHG or FHpHG-Tent5a, probed for the HA tag. ACTB was detected to confirm equal loading. Molecular weight markers (kDa) are shown on the right.

B. PCR gel of genomic DNA from $Tent5a^{WT/null}$, $Tent5a^{WT/WT}$, and $Tent5a^{null/null}$ MEF cells using primers specific for the $Tent5a^{null}$ (upper panel) and $Tent5a^{WT}$ (lower panel) alleles. Molecular weight markers (bp) are shown on the right. The expected band sizes are 161 and 279 bp for the wild-type and null alleles, respectively.

C. Bar plot showing *Tent5a* mRNA levels in $Tent5a^{WT}$ and $Tent5a^{null}$ MEF cell lines, as measured by qPCR. Results are presented as mean ± SD of three biological replicates. Statistical significance was determined by Student's t-test: ****, $p < 0.0001$.

Figure S3

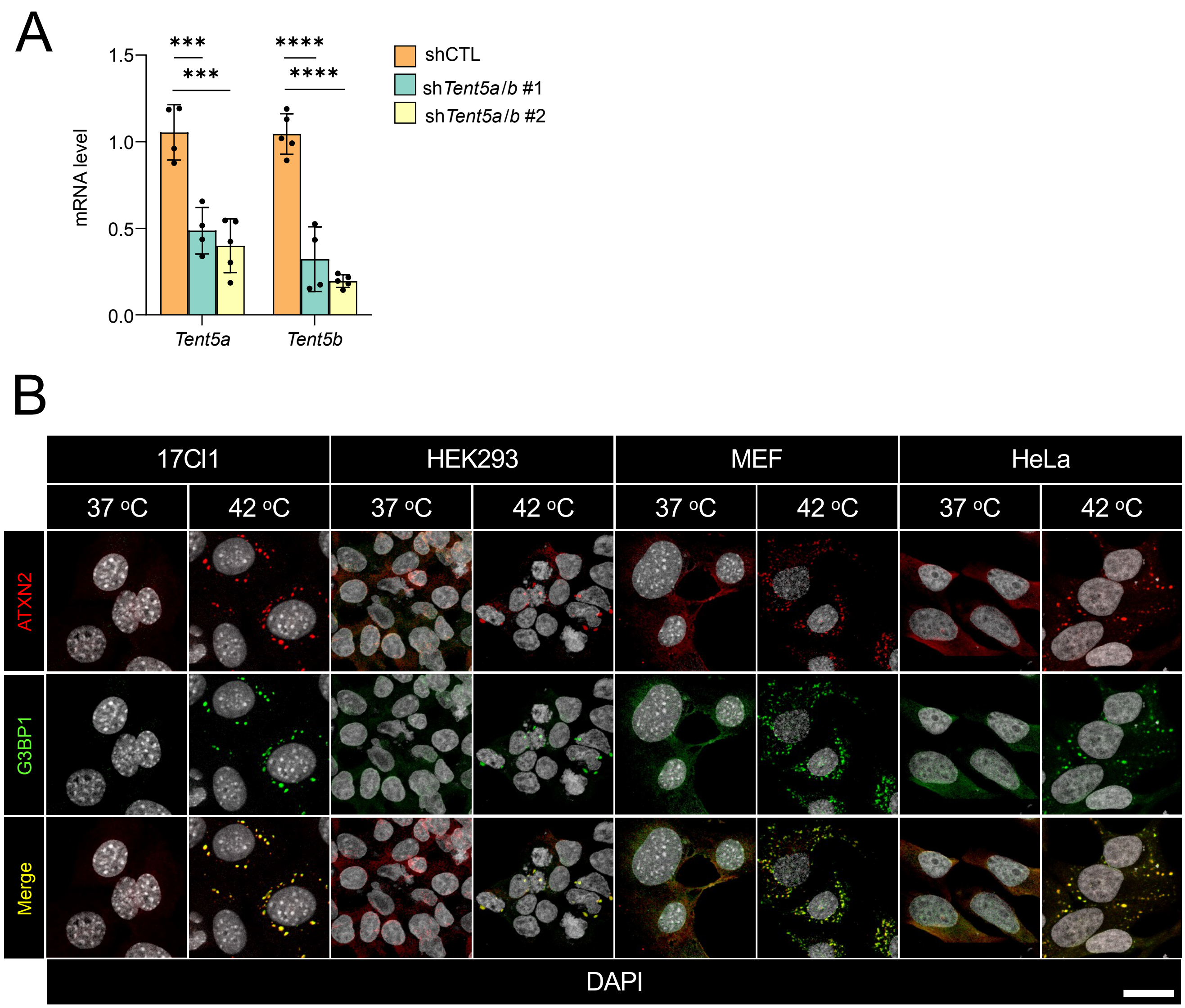

Figure S3. ATXN2 localizes to G3BP1-positive granules.
A. Bar plot of Tent5a and Tent5b mRNA levels in 17Cl1 cells infected with lentivirus expressing control shRNA (shCTL) or two combinations of shRNAs targeting Tent5a and Tent5b (shTent5a/b #1 and shTent5a/b #2). Data represent mean ± SD from four to five biological replicates. Statistical significance was determined by unpaired two-tailed Student's t-test comparing each condition to shCTL. ***, $p < 0.001$; ****, $p < 0.0001$.
B. Confocal images of 17Cl1, HEK293, MEF, and HeLa cells following 1-hour incubation at 37 °C or 42 °C. ATXN2 and G3BP1 were detected using specific antibodies and are shown in red and green, respectively. Nuclei were counterstained with DAPI (white). ATXN2, G3BP1, and the merge (yellow) are shown. The experiment was repeated three times. Scale bar, 20 μm.

Figure S4

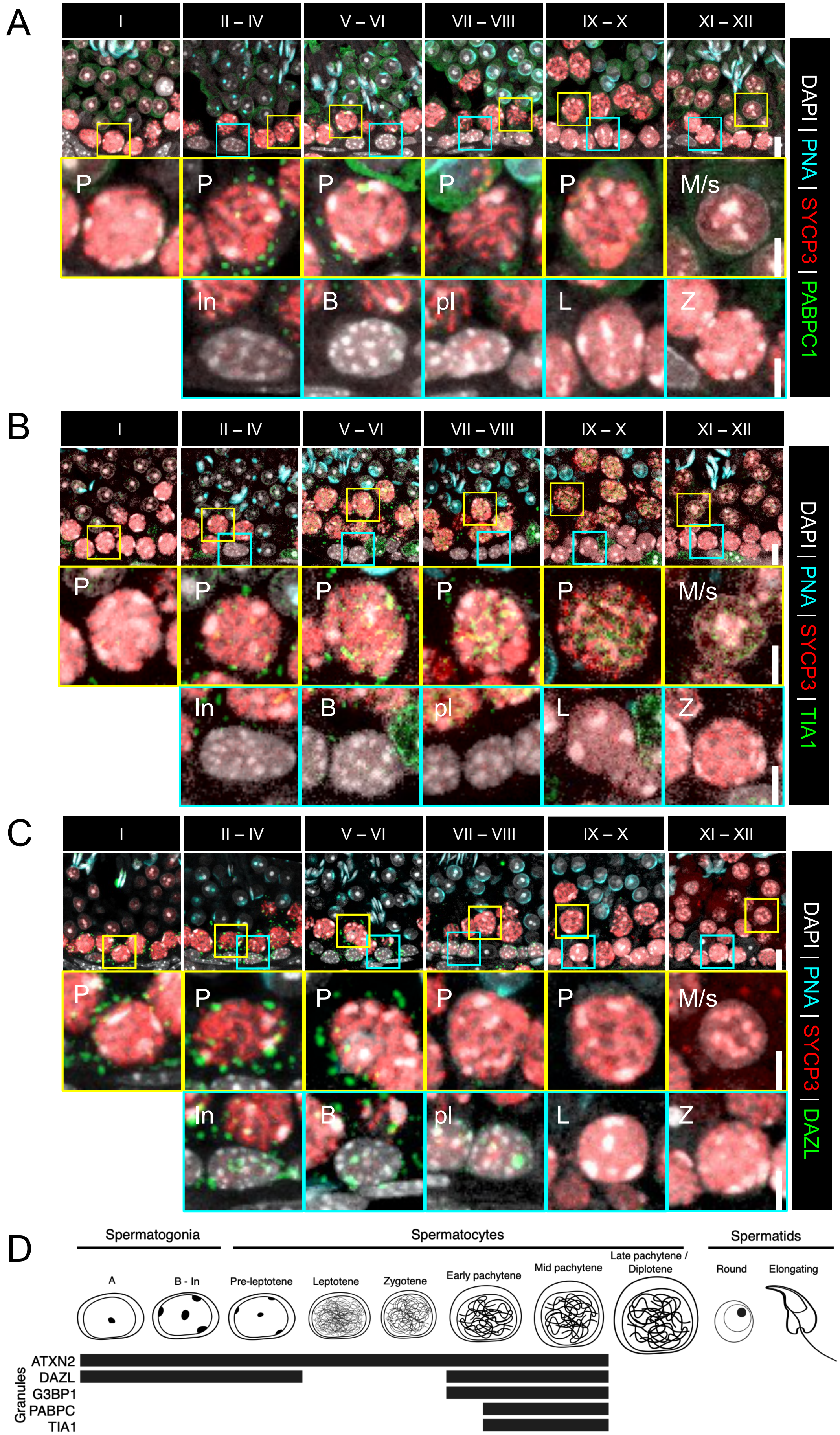

A
I
II – IV
V – VI
VII – VIII
IX – X
XI – XII
P
M/s
In
B
pl
L
Z
DAPI | PNA | SYCP3 | PABPC1
B
DAPI | PNA | SYCP3 | TIA1
C
DAPI | PNA | SYCP3 | DAZL
D
Spermatogonia
Spermatocytes
Spermatids
A
B - In
Pre-leptotene
Leptotene
Zygotene
Early pachytene
Mid pachytene
Late pachytene / Diplotene
Round
Elongating
Granules
ATXN2
DAZL
G3BP1
PABPC
TIA1

Figure S4. PABPC1, TIA1, and DAZL form stress granules in early and mid-pachytene SPCs.

A-C. Confocal images of testicular cross-sections from WT mice following 20 minutes of scrotal exposure to 42°C. Acrosomes and SPCs were labeled with PNA (cyan) and SYCP3 (red), respectively. SG components PABPC1 (**A**), TIA1 (**B**), and DAZL (**C**) are shown in green. Representative seminiferous tubule stages I, II-IV, V-VI, VII-VIII, IX-X, and XI-XII are displayed. Yellow insets show pachytene SPCs and blue insets show differentiating SPG and pre-pachytene SPCs. P, pachytene SPC; M/s, metaphase or secondary SPC; In, intermediate SPG; B, B SPG; pl, preleptotene SPC; L, leptotene SPC; and Z, zygotene SPC. Scale bars = 10 μm (main images) and 5 μm (insets). Confocal experiments (**A-C**) were repeated using two different mice.

D. Schematic representation of adult spermatogenesis showing the distribution of SG components ATXN2, G3BP1, TIA1, DAZL, and PABPC1 across male germ cell populations.

Figure S5

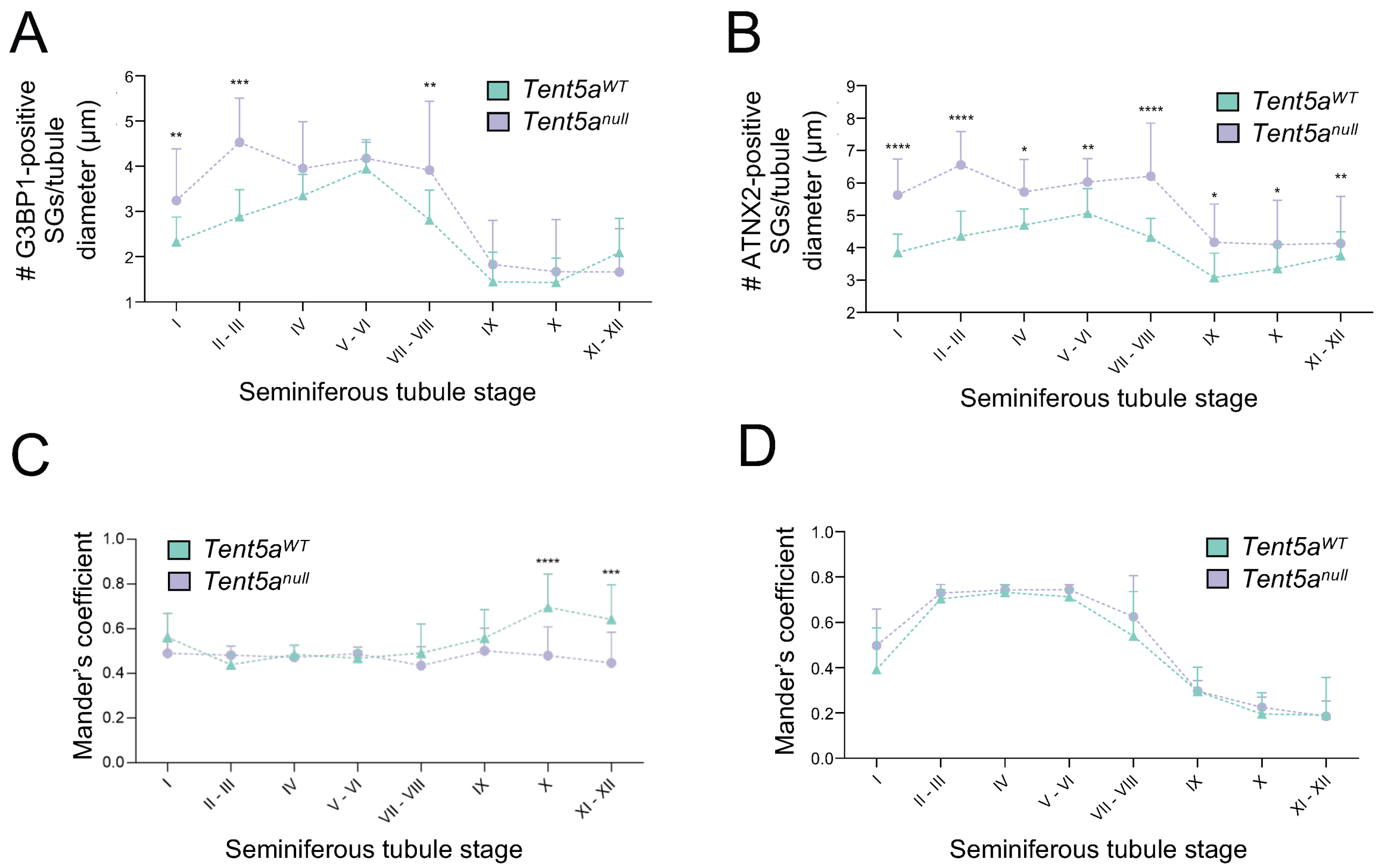

Figure S5. Quantitative analysis of ATXN2 and G3BP1-positive granules through different stages of the seminiferous cycle.

A and B. Line graphs showing the number of G3BP1-positive (**A**) and ATXN2-positive (**B**) SGs across seminiferous tubules from *Tent5a*^{WT} and *Tent5a*^{null} mice after 20 minutes of scrotal heat exposure. SG abundance was normalized to tubule diameter (μm) to account for variation in tubule size.

C and D. Line graphs showing Mander's overlap coefficient representing the fraction of ATXN2 signal overlapping with G3BP1 (**C**) and the fraction of G3BP1 overlapping with ATXN2 (**D**) across all stages of the seminiferous epithelial cycle in *Tent5a*^{WT} (green) and *Tent5a*^{null} (purple) mice following 20 minutes of scrotal heat exposure at 42 °C.

The replicate values of 134 and 123 seminiferous tubule cross sections from *Tent5a*^{WT} and *Tent5a*^{null} testes, respectively are shown (**A-D**). Student's t-test was used to compare each experimental condition to the control condition, *, $p < 0.05$; **, $p < 0.01$; ***, $p < 0.001$; ****, $p < 0.0001$; ns, $p > 0.05$.

Figure S6

A

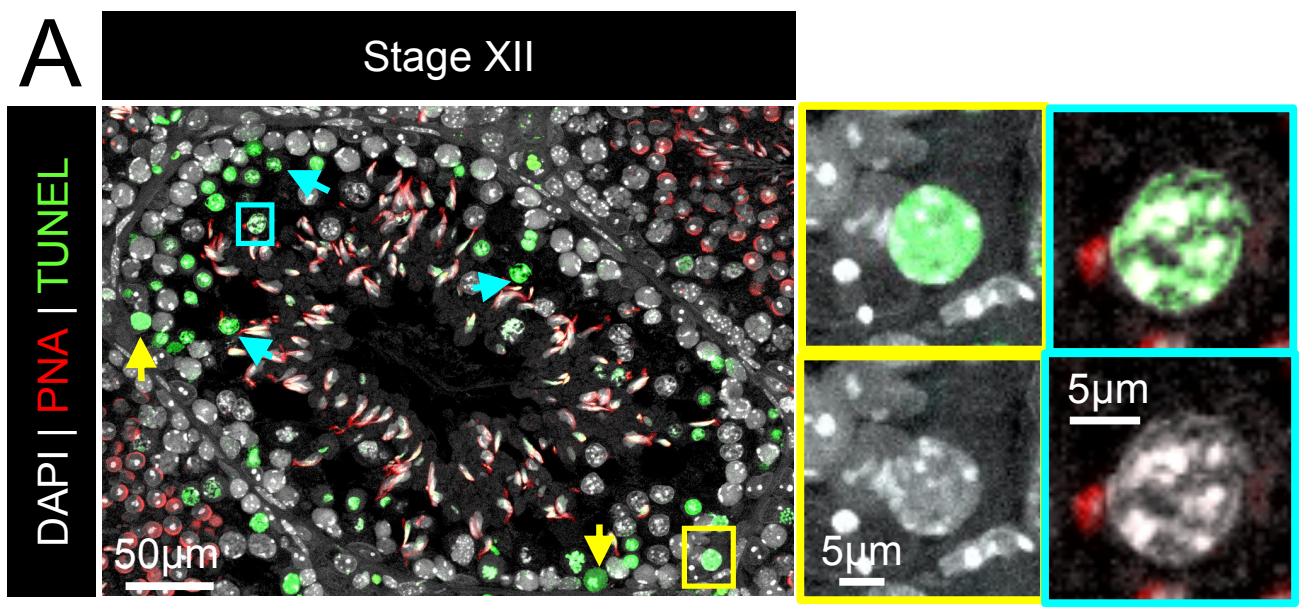

F

	observed litters	missed litters
Tent5a/c$^{fl/fl}$	25	30
Tent5a/c$^{fl/fl}$; *Ddx4Cre*^{ERT2}	49	60
33 °C	37	43
42 °C	37	47

B C D

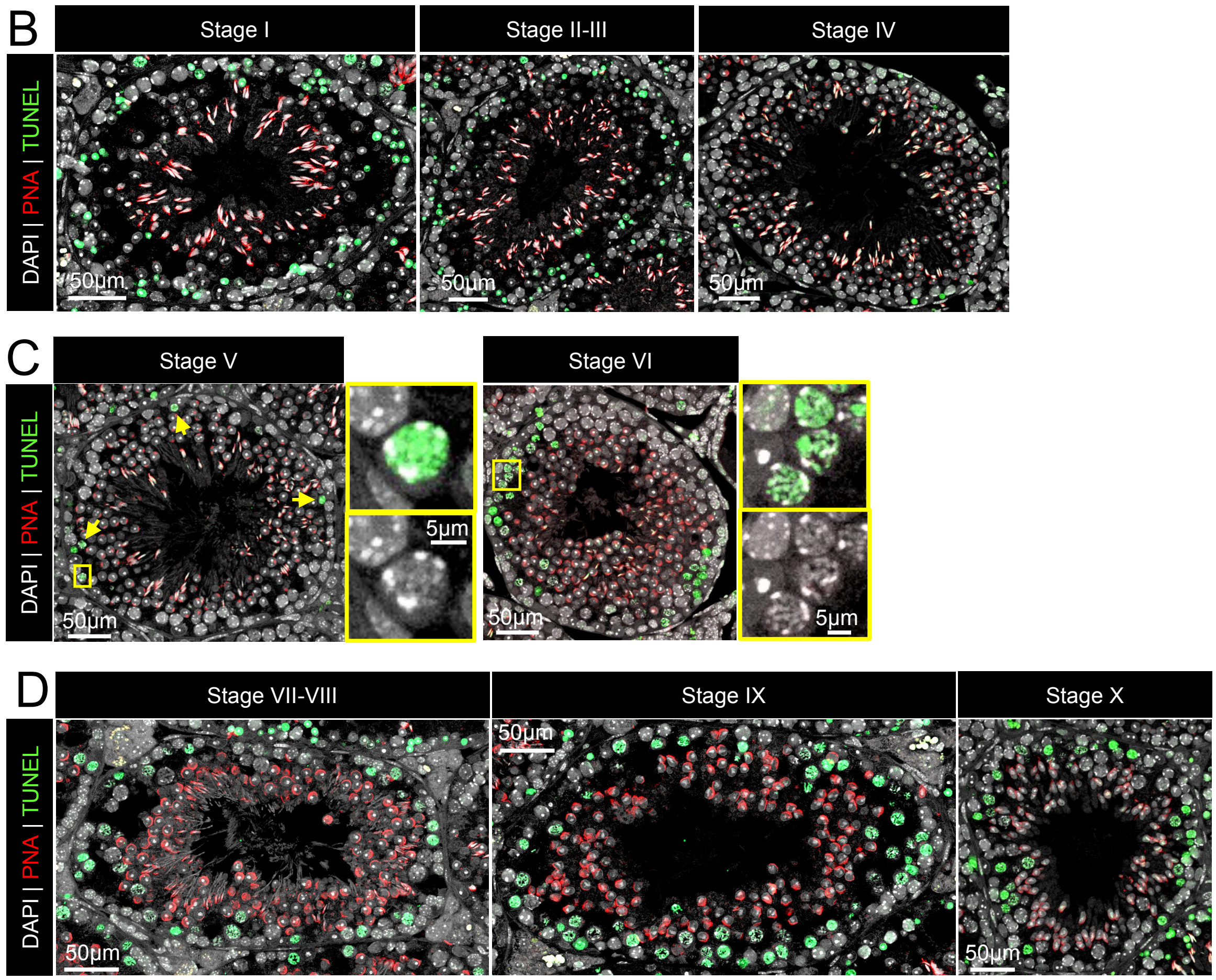

E

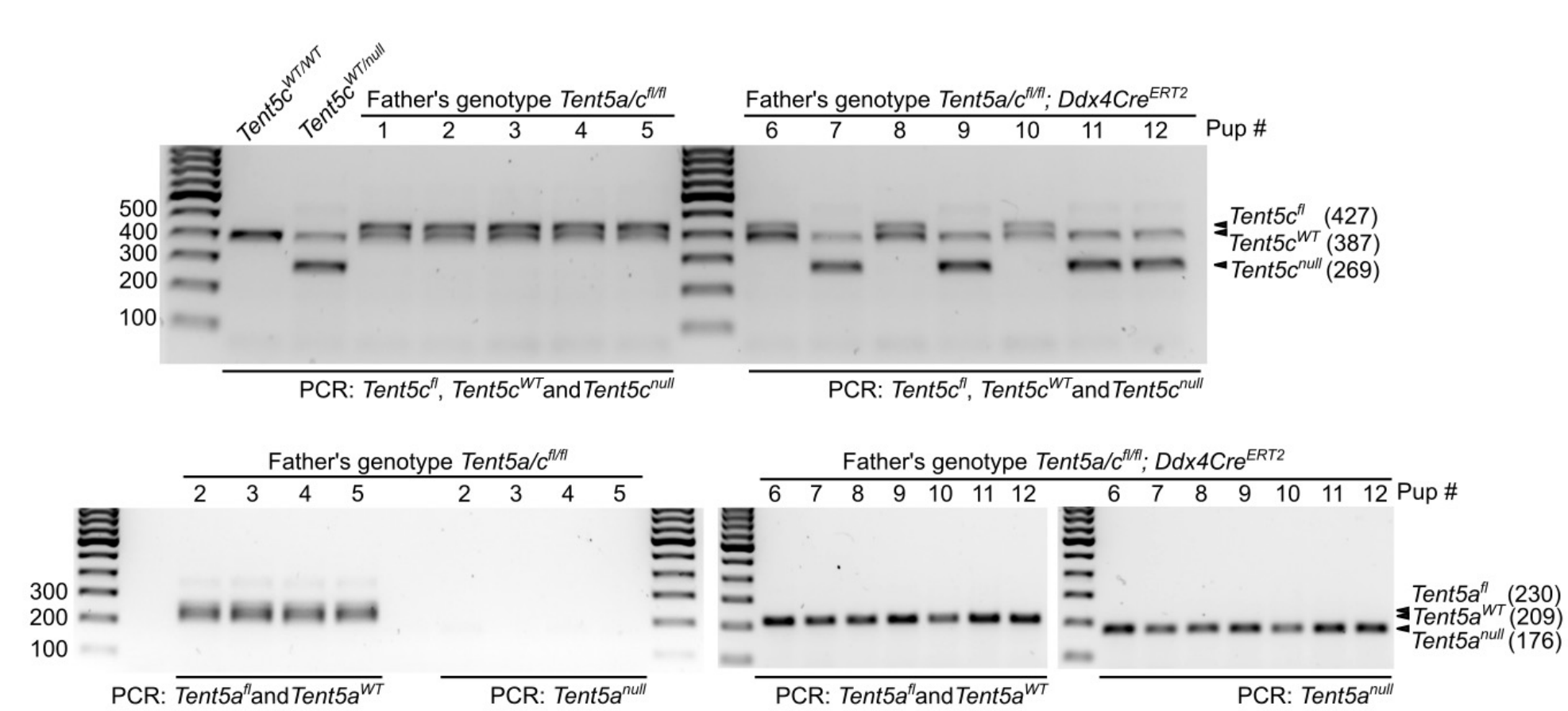

Figure S6. Loss of Tent5a confers protective effects against heat exposure.

A, B, C, and D. Confocal images of testicular cross-sections from *Tent5a*^{WT} mice 6 hours after scrotal exposure to 42 °C. Stages XII (**A**), I–IV (**B**), V–VI (**C**), and VII–X (**D**) are shown. Apoptotic cells were labeled by TUNEL (green), acrosomes were stained with PNA (red), and nuclei were counterstained with DAPI (white). Insets display TUNEL and DAPI merge (top), and DAPI only (bottom). Scale bars: 50 μm (main panels), 5 μm (insets). The experiments (**A-D**) were repeated with 5 mice.

E. Gel electrophoresis images showing genotyping results from a litter sired by a tamoxifen-treated *Tent5a/c*$^{fl/fl}$ male mated with a WT female (left runs; n=5 pups; pup # 1 to 5) and from a litter sired by a tamoxifen-treated *Tent5a/c*$^{fl/fl}$*; Ddx4Cre*^{ERT2} male mated with a WT female (right runs; n=7 pups; pup # 6 to 12) using allele-specific primers for the *Tent5a* and *Tent5c* loci. PCR products corresponding to the WT, floxed, and null alleles are shown. The molecular sizes in base pairs are indicated on the left of the gels. The position of the expected bands for the different alleles, together with their size in base pairs, are shown on the right. The alleles detected by the different PCR strategies are indicated below each section of the gel.

F. Table showing the observed and missed number of litters (i.e., maximum number of possible litters during the fertility trial minus the observed number of litters) according to the different genotypes (*Tent5a/c*$^{fl/fl}$ mice or *Tent5a/c*$^{fl/fl}$*; Ddx4Cre*^{ERT2}) and exposures (33 °C or 42 °C).

Figure S7

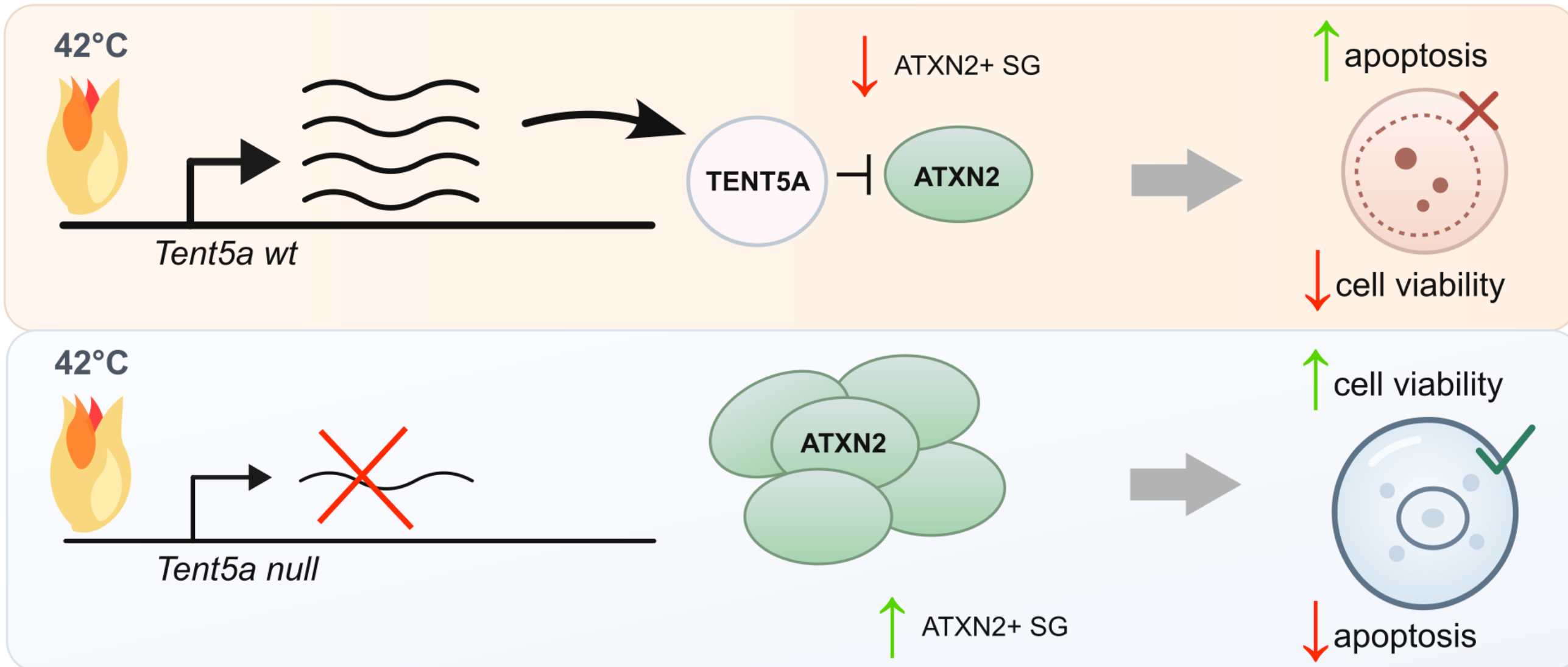

Figure S7. TENT5A-mediated downregulation of ATXN2-positive stress granules during heat stress promotes apoptosis.
Top: *Tent5a* wt cells show elevated expression of Tent5a upon heat exposure. Increased levels of TENT5A result in the downregulation of ATXN2 and ATXN2-associated stress granules, leading to apoptosis and cell death. Bottom: *Tent5a* null cells show increased ATXN2 levels and stress granule formation, which reduces apoptosis.
