# Supplementary material for: The TENT5A-ATXN2 axis modulates germline and somatic cell survival during heat stress
Source: Cell Death Dis. 2026 Jul 14;17(1):871. doi: 10.1038/s41419-026-09086-1 (PMC13646263; doi:10.1038/s41419-026-09086-1)

S2A

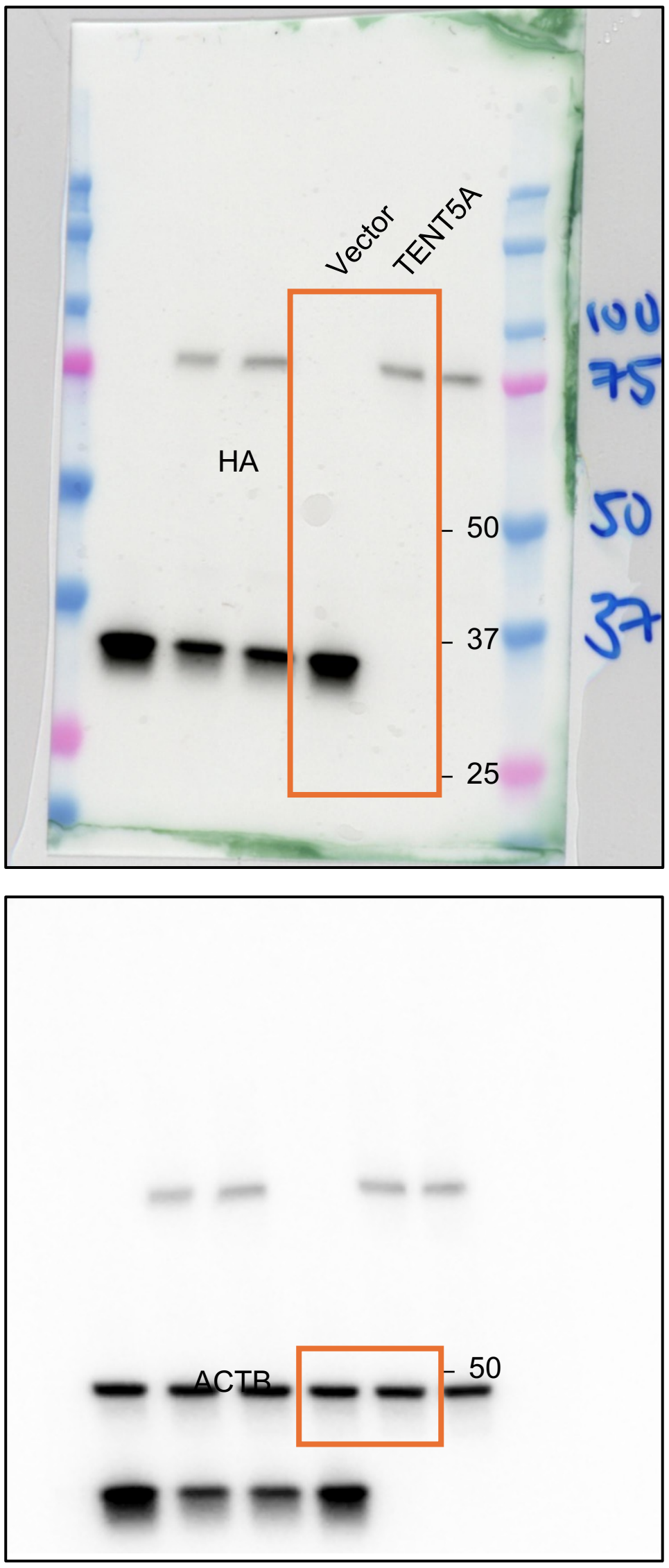
Vector
TENT5A
100
75
HA
50
37
25
ACTB
50

3G

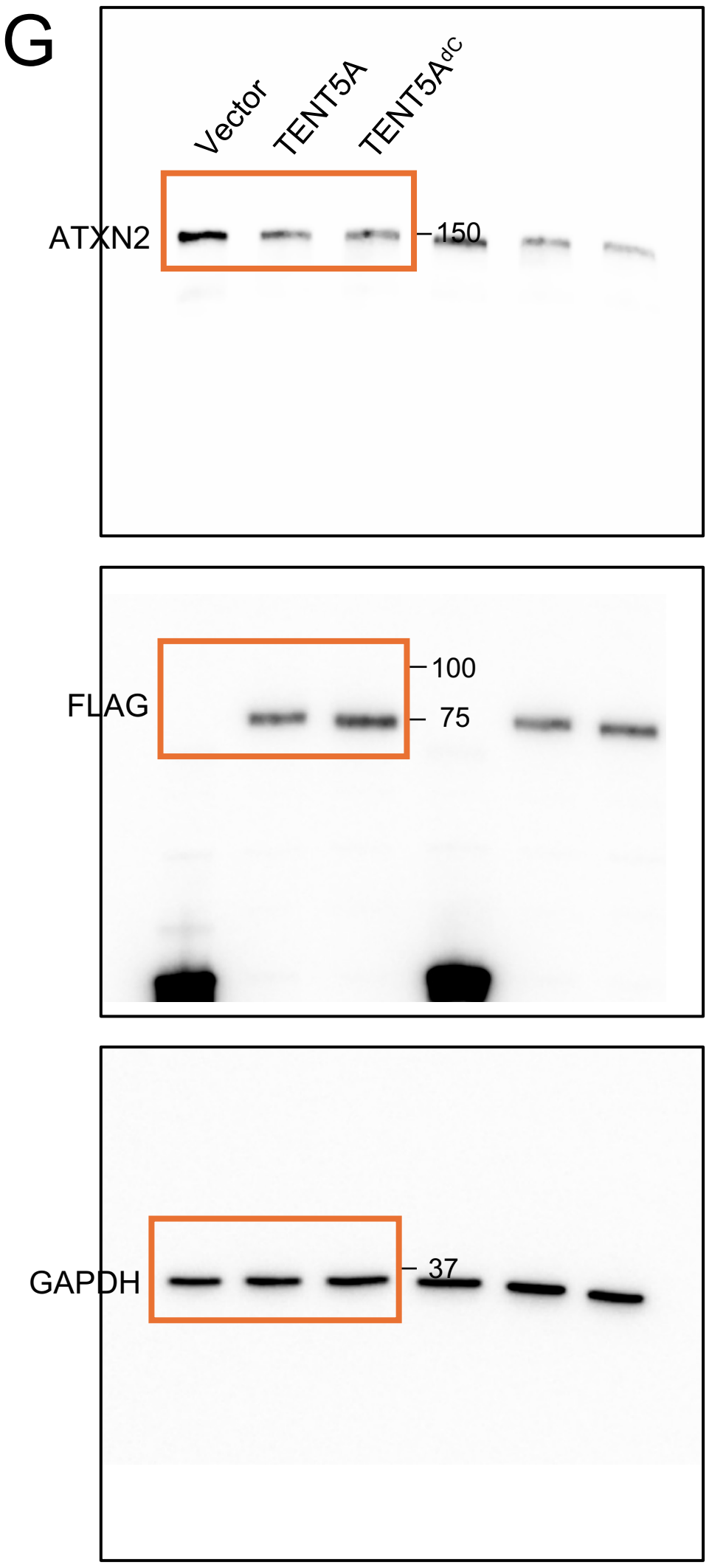
Vector
TENT5A
$TENT5A^{dC}$
ATXN2
150
FLAG
100
75
GAPDH
37

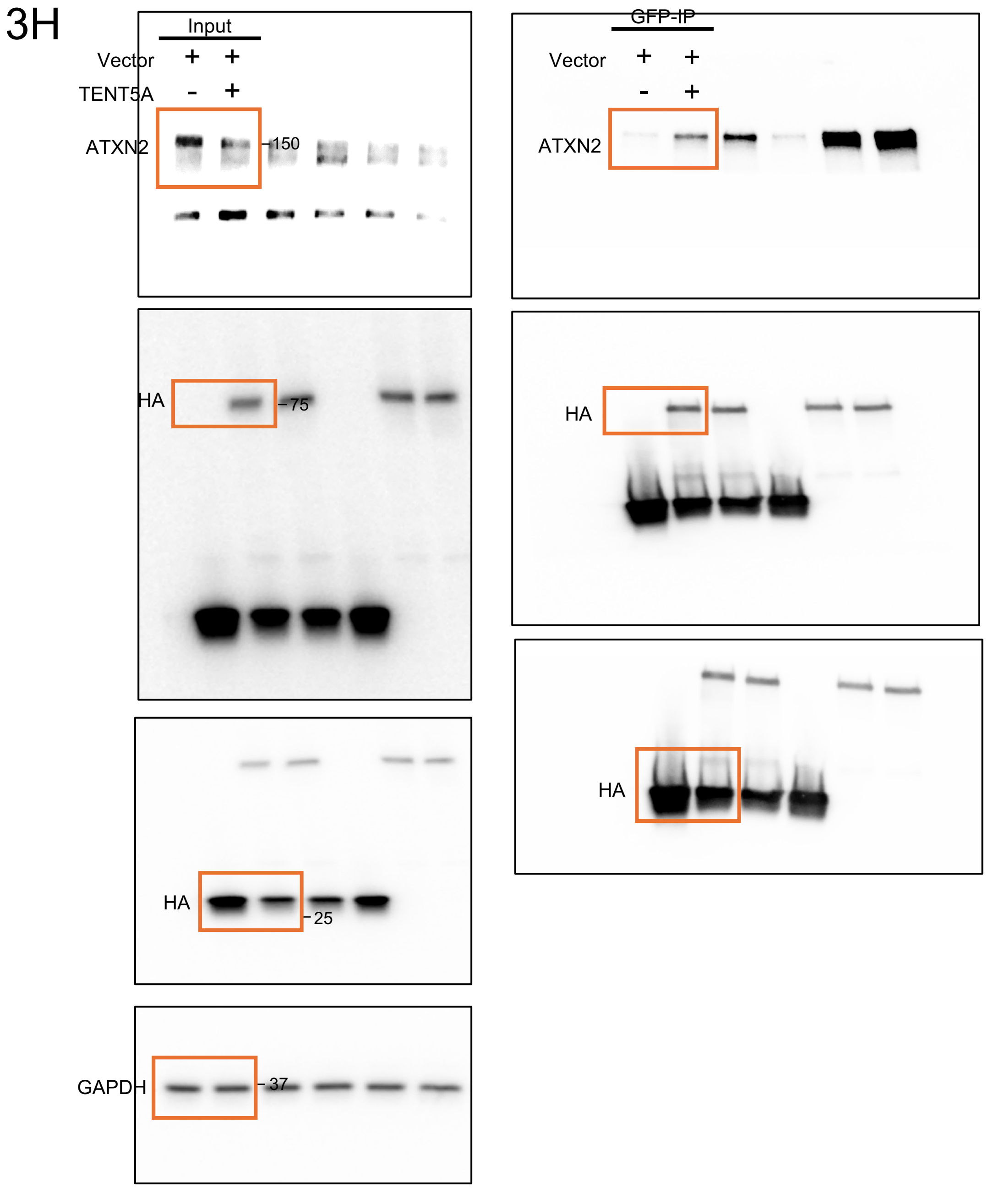
3H
Input
Vector + +
TENT5A - +
ATXN2
150
HA
75
HA
25
GAPDH
37
GFP-IP
Vector + +
- +
ATXN2
HA
HA

4E

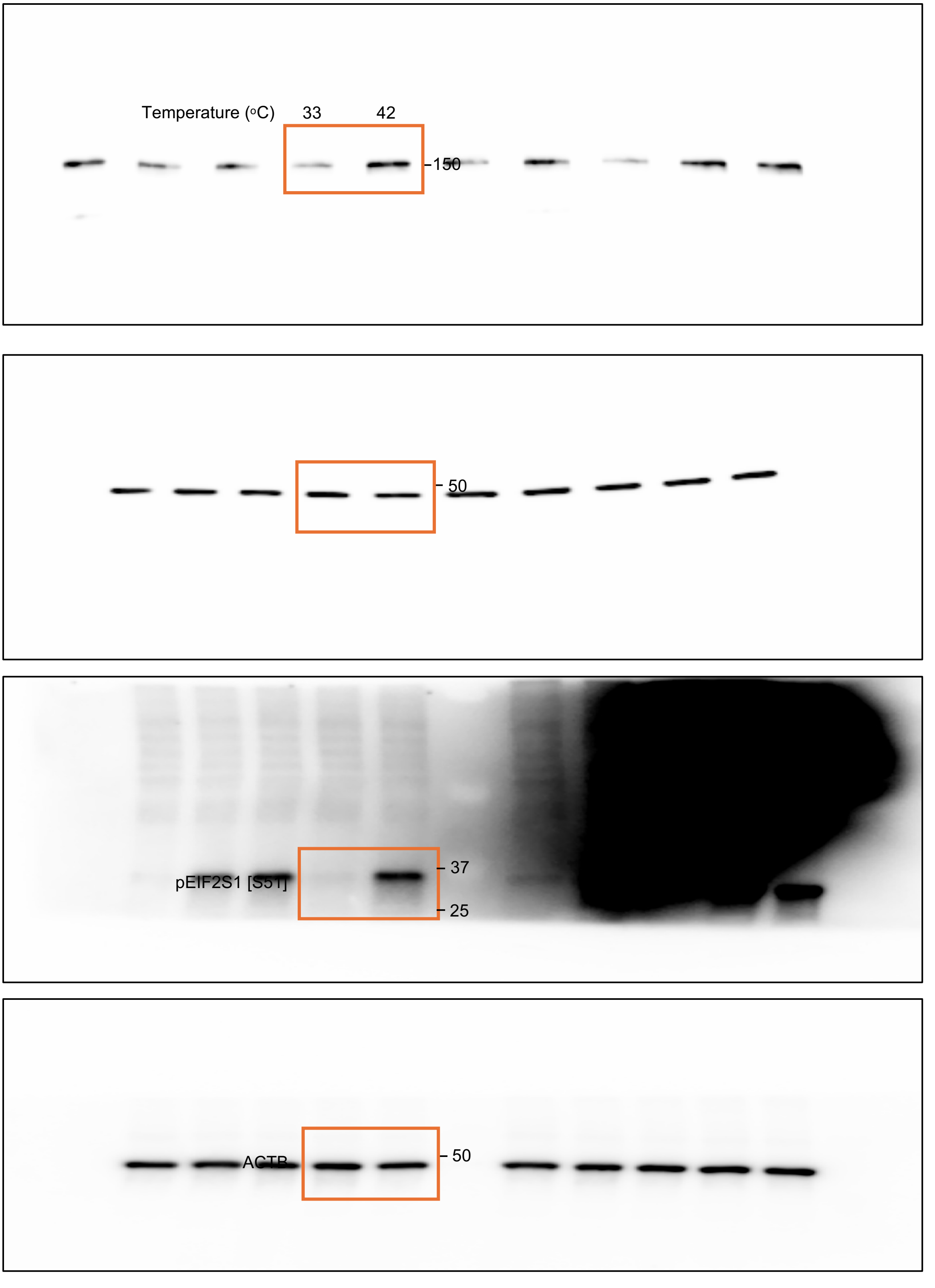

Supplement: Supplementary file 2 [file 41419_2026_9086_MOESM4_ESM.pdf]
